# Pharmacokinetic Simulation and Area under the Curve Estimation of Drugs Subject to Enterohepatic Circulation

**DOI:** 10.3390/pharmaceutics16081044

**Published:** 2024-08-06

**Authors:** Melchor Alpízar, José de Jesús Reséndiz, Elisa García Martínez, Sanyog Dwivedi, Miguel Alejandro Trejo

**Affiliations:** Specialized Centre for Diabetes, Obesity and Prevention of Cardiovascular Diseases (CEDOPEC) & Bioequivalence Studies (E-BIOS-CEDOPEC), Mexico City 11650, Mexico; jresendiz@cedopec.com (J.d.J.R.); egarcia@cedopec.com (E.G.M.); sanyogdwivedi@outlook.com (S.D.)

**Keywords:** enterohepatic circulation (EHC), pharmacokinetics, mathematical modeling, therapeutic drug monitoring (TDM), area under the curve (AUC)

## Abstract

Enterohepatic circulation (EHC) is a complex process where drugs undergo secretion and reabsorption from the intestinal lumen multiple times, resulting in pharmacokinetic profiles with multiple peaks. The impact of EHC on area under the curve (AUC) has been a topic of extensive debate, questioning the suitability of conventional AUC estimation methods. Moreover, a universal model for accurately estimating AUC in EHC scenarios is lacking. To address this gap, we conducted a simulation study evaluating five empirical models under various sampling strategies to assess their performance in AUC estimation. Our results identify the most suitable model for EHC scenarios and underscore the critical role of meal-based sampling strategies in accurate AUC estimation. Additionally, we demonstrate that while the trapezoidal method performs comparably to other models with a large number of samples, alternative models are essential when sample numbers are limited. These findings not only illuminate how EHC influences AUC but also pave the way for the application of empirical models in real-world drug studies.

## 1. Introduction

**Figure 1:**
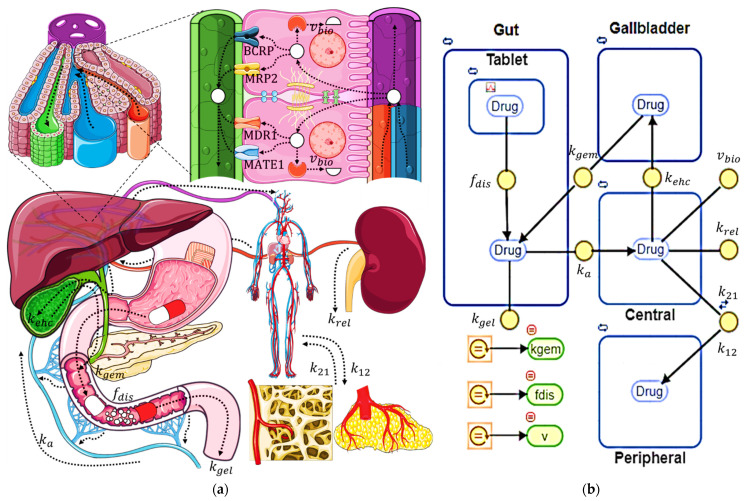
(**a**) Kinetic processes present in the EHC. Additional kinetic processes may occur, such as fecal elimination of a fraction of the drug secreted in the bile or not absorbed, renal elimination, distribution to peripheral tissues, hepatic biotransformation, and release of the pharmaceutical form for drugs for extravascular administration; (**b**) scheme of the compartmental model implemented for the simulation of the EHC. Rectangles with rounded corners represent the model compartments. The yellow circles represent the drug transfer parameters between the compartments, the majority being first-order constants. Green ellipses represent drug transfer parameters that are defined by an equation.

Enterohepatic circulation (EHC) represents a dynamic kinetic process integral to the pharmacokinetics of certain drugs, delineating a crucial pathway within the body’s metabolic landscape [1]. This intricate cycle commences with drug absorption from the intestine, where a portion of the dissolved drug traverses through the portal system to reach the hepatic lobules. Here, within the intricate architecture of the liver, the drug navigates through branches of the hepatic artery and portal vein, eventually circulating to the central vein. Along this journey, a fraction of the drug diffuses through the sinusoidal walls, finding its way into hepatocytes [2,3]. Once within these hepatic cells, the drug may undergo active transportation, facilitated by an array of pumps, such as BCRP, MATE1, MDR3, or MRP2, towards the bile canaliculi, ultimately contributing to bile formation [2,3]. Subsequently, bile, laden with the drug, travels through the bile ducts to the gallbladder, where it awaits the signal for release prompted by food intake and the hormone cholecystokinin [4,5]. The gallbladder then empties its contents, a process spanning approximately 60 min, through the sphincter of Oddi into the duodenum [6,7,8]. Within the duodenum, the drug dissolved in bile may undergo reabsorption, initiating a new cycle of circulation. Figure 1a elucidates these intricate processes alongside parallel kinetic phenomena.

In studying EHC, researchers analyze time versus plasma drug concentration curves to uncover the dynamic interplay between absorption, distribution, metabolism, and excretion, revealing peaks and troughs corresponding to distinct phases of the enterohepatic cycle. Despite offering valuable insights, such analysis is challenged by variability in physiology, drug properties, and environmental factors, along with practical considerations like sampling frequency and timing. Understanding EHC is crucial in drug studies, as it profoundly influences pharmacokinetic parameters essential for determining pharmaceutical efficacy and safety. Consequently, accurately characterizing these plasma profile fluctuations poses a significant challenge in EHC studies, necessitating the development of precise models.

The journey to model EHC began with compartmental models [9], conceptualizing the intestine and organism as discrete compartments with drug transfer occurring continuously [10] or discontinuously [11,12]. Over time, these models evolved in complexity, incorporating additional compartments to account for the gallbladder. Dynamics governing the release of bile into the intestine were then modeled with various kinetics, including continuous [13], single [14,15,16,17,18,19], or multiple, the latter encompassing models utilizing switch-type [20,21,22,23,24,25,26], sigmoid [27], or sinusoidal [28,29] functions. This evolution facilitated a deeper comprehension of EHC and its ramifications on drug pharmacokinetics.

Physiologically based pharmacokinetic models represent the pinnacle of complexity within EHC modeling, treating organs and tissues as distinct entities with specific properties for both organism and drug [30,31,32,33]. However, despite their increasing prevalence, their translation into clinical therapeutics remains limited [34,35].

Alternatively, empirical mathematical functions, devoid of physiological or compartmental underpinnings, have been employed in EHC modeling [36]. The pioneering model in EHC employed two gamma functions to fit the plasma mycophenolic acid profile, while another model utilized the sum of two Gaussian functions to depict the plasma profile of simvastatin. Demonstrated feasibility extends these models beyond two peaks by incorporating additional terms [37,38,39]. While these models may not elucidate EHC’s underlying processes, they align with experimental data and capture the secondary peak characteristics of EHC. Consequently, they have found widespread implementation in clinical settings as valuable tools in therapeutic drug monitoring (TDM).

Understanding the magnitude of EHC for drugs assumes paramount importance, as it significantly influences key pharmacokinetic parameters such as clearance (Cl), volume of distribution (Vd), half-life plasma ($t_{1/2}$), and AUC [40]. Precisely, the AUC is frequently used in TDM for dose adjustment in drugs with a narrow therapeutic range [41]. Sampling times and the number of samples are a limiting factor in TDM that requires restricting the sampling strategy. On the other hand, the AUC is an essential parameter in bioequivalence studies, and it has been argued that the conventional trapezoidal method, used to calculate the AUC, probably overestimates its value when EHC exists.

The debate over whether empirical models offer advantages over traditional methods, like the trapezoidal rule, in estimating AUC remains unresolved. Thus, this study aims to evaluate the efficacy of several empirical models in characterizing time–concentration profiles of hypothetical and real drugs sharing the trait of undergoing EHC. This evaluation, grounded in simulations of various pharmacokinetic scenarios, aims to shed light on the potential of empirical models in understanding and quantifying EHC’s impact from both conventional and limited sampling perspectives.

## 2. Materials and Methods

A compartmental model and a set of empirical models were developed. The compartmental model was used to perform simulations of different pharmacokinetic scenarios; the empirical models were used to estimate the AUC of these simulations.

### 2.1. Compartmental Pharmacokinetic Model

The compartmental model was built considering the kinetic processes involved in EHC, in addition to those that can occur in parallel and that contribute to global pharmacokinetics; see Figure 1a.

The behavior of the EHC phenomenon is schematized in Figure 1b and is given the following system of differential equations:

(1) $$\left\{\begin{array}{c} \frac{dD_{C}}{dt}=k_{a}D_{G}+k_{21}D_{P}-k_{12}D_{C}-k_{ehc}D_{C}-k_{rel}D_{C}-v_{bio}\\ \frac{dD_{P}}{dt}=k_{12}D_{C}-k_{21}D_{P}\\ \frac{dD_{G}}{dt}=f_{dis}D_{S}+k_{gem}D_{B}-k_{a}D_{G}-k_{gel}D_{G}\\ \frac{dD_{S}}{dt}=-f_{dis}D_{S}\\ \frac{dD_{B}}{dt}=k_{ehc}D_{C}-k_{gem}D_{B} \end{array}\right.$$

where $D_{x}$ is the concentration of drug and whose subscript indicates the site in which it is located ($D_{c}$ central compartment; $D_{P}$ peripheral; $D_{G}$ intestinal; $D_{B}$ biliary; $D_{S}$ in the solid pharmaceutical form). Except for the parameters $f_{dis}$, $k_{gem}$, and $v_{bio}$, which are set as parameters as a function of time or drug concentration, most of the parameters in this system of equations are constant during the simulation.

Fraction of dissolved dose of solid dosage form ($f_{dis}$) is one of the time-dependent parameters and was simulated with the Weibull function in Equation (2). This is a function without a physicochemical basis widely used in modeling drug dissolution [42], and it is expressed as follows:

(2) $$\begin{array}{c} f_{dis}\left(t\right)=1-e^{-{\left(\frac{t-t_{lag}}{k_{d}}\right)}^{a}} \end{array}$$

where $t_{lag}$ corresponds to the time it takes to start the dissolution process, $k_{d}$ is the scale parameter that determines the fraction of drug released from the formulation, and a is the shape parameter that characterizes the curve as exponential (a=1), sigmoid (a>1), or parabolic (a<1).

Emptying of the gallbladder ($k_{gem}$) is a time-dependent parameter; continuous function, given by Equation (3), was used to simulate emptying of the gallbladder and allows the generation of several emptying cycles distributed over a period of 24 h; it is expressed in the following way:

(3) $$\begin{array}{c} k_{gem}\left(t\right)=k_{max}\overset{n}{\underset{i=1}{\sum }}e^{-b{\left(\sin\frac{\pi \left(t-c_{i}\right)}{24}\right)}^{2}} \end{array}$$

where the parameter $k_{max}$ is the maximum emptying speed of the gallbladder, the parameter b is related to the duration of each of the emptying cycles, and the parameter $c_{i}$ allows the establishment of the moments in which the emptying of gallbladder occurs for each of the EHC cycles according to meal times (established as hours elapsed after drug intake).

The last non-constant parameter $v_{bio}$ is a function of the drug concentration in the central compartment and represents the biotransformation rate of the drug. For its calculation, the Michaelis–Menten equation, Equation (4), is used. This equation allows us to consider the effects of the competition that may exist between biotransformation and other processes that the drug may have (bile transfer, transfer to the peripheral compartment, or renal elimination), and it is expressed as follows:

(4) $$\begin{array}{c} v_{bio}=\frac{v_{max}D_{C}}{k_{m}+D_{C}} \end{array}$$

where $k_{m}$ is the Michaelis–Menten constant, and $v_{max}$ is the maximum biotransformation rate.

To complete the analysis, the percentage of drugs within the central compartment undergoing enterohepatic circulation was calculated. This percentage is not necessarily constant over time; Equation (5) allows the calculation of its temporal fluctuation, while Equation (6) calculates its theoretical maximum value.

(5) $$\begin{array}{c} EHC\%=\frac{k_{ehc}}{k_{ehc}+k_{rel}+k_{12}+v_{bio}}\times 100\% \end{array}$$

(6) $$\begin{array}{c} EHC_{max}\%=\frac{k_{ehc}}{k_{ehc}+k_{rel}+k_{12}}\times 100\% \end{array}$$

### 2.2. Compartmental Model Assumptions

The compartmental model was developed with the objective of representing the EHC and some parallel pharmacokinetic processes in the most realistic way possible; however, to simplify the analysis, some reasonable assumptions are made:

- Compartments are homogeneous, so drug concentration within each of the compartments reaches an instantaneous kinetic equilibrium;
- Liver is located within the central compartment, so there is an instantaneous kinetic equilibrium between plasma and intrahepatic drug concentrations;
- Elimination by routes other than renal, fecal, or biotransformation is insignificant;
- Fraction of drug that is biotransformed does not need to be reconverted to the original drug to undergo EHC, as this usually happens under the presence of microbiota glucuronidases;
- Elimination by biotransformation occurs through a single pathway involving a single enzyme;
- Drug dissolved in the bile is excreted to the intestinal compartment only from the bile compartment, so direct excretion from the central compartment is non-existent;
- Fraction of drug dissolved, transfer of drug from the gallbladder to the intestine, and rate of biotransformation can be realistically represented by equations, while the rest of the parameters follow first-order kinetics that do not consider changes in transport rates between compartments due to physiological phenomena.

Finally, during simulations, it was assumed that the gallbladder is only activated by meals three times during a 24-hour period; at other times, its emptying is null.

### 2.3. Pharmacokinetic Simulations

The compartmental model was implemented through the SimBiology tool of MATLAB version R2021a. Drug concentrations versus time were simulated with the sundials solvertype. The description, units, and specific values of model parameters are shown in Table 1. These values were proposed to have a starting model for subsequent scenarios.

Data shown in Table 1 correspond to those of the standard model from which the values of one parameter were varied at a time to generate 18 different scenarios. These scenarios are grouped into pairs that differ in whether the value of the modified parameter is greater or less than its value in the standard model. Table 2 shows the description, the modified parameter, and its value in each scenario.

**Table 1:** Parameters of the standard model.

Parameter	Units	Value	Description
$k_{a}$	h^−1^	0.9	Intestinal absorption rate
$k_{rel}$	h^−1^	0.1	Renal elimination rate
$k_{gel}$	h^−1^	0.1	Fecal elimination rate
$f_{dis}$	-	See Equation (2)	Dissolved drug fraction
$k_{d}$	h^−1^	0.5	Scale parameter of the Weibull function
$t_{lag}$	h	0	Weibull function delay time
a	-	1	Shape parameter of the Weibull function
$k_{gem}$	h^−1^	See Equation (3)	Rate of transfer from gallbladder to intestine
$k_{max}$	h^−1^	3.0	Maximum transfer rate from gallbladder to intestine
b	-	300	Biliary emptying cycle duration parameter
$c_{i}$	-	5, 11, 17	Times of maximum gallbladder emptying rate
$k_{12}$	h^−1^	0	Transfer rate from central to peripheral compartment
$k_{21}$	h^−1^	0	Transfer rate from peripheral to central compartment
$v_{bio}$	mg/h	See Equation (4)	Instantaneous biotransformation rate
$v_{max}$	mg/h	0.1	Maximum biotransformation rate
$k_{m}$	mg/L	See Table 3	Michaelis–Menten constant for biotransformation
$k_{ehc}$	h^−1^	See Table 3	Transfer rate from the central compartment to the gallbladder

**Table 2:** Modified parameters in pharmacokinetic scenarios.

Scenarios	Parameter	Value	$k_{m}$ ^a^	$k_{ehc}$ ^a^	Description
$S_{1}$	$k_{a}$	1.5	I	A	Rapid absorption in the intestinal compartment
$S_{2}$		0.3	I	A	Slow absorption in the intestinal compartment
$S_{3}$	$k_{rel}$	0.5	II	B	Rapid renal elimination from central compartment
$S_{4}$		0.02	III	C	Slow renal elimination from central compartment
$S_{5}$	$k_{gel}$	0.5	I	A	Rapid elimination from intestinal compartment
$S_{6}$		0.02	I	A	Slow elimination from intestinal compartment
$S_{7}$	$t_{lag}$	0.0	I	A	Drug release without delay time
$S_{8}$		2.0	I	A	Time-delayed drug release
$S_{9}$	a	0.5	I	A	Parabolic drug release
$S_{10}$		2.0	I	A	Sigmoidal drug release
$S_{11}$	$k_{max}$	6.0	I	A	Rapid gallbladder emptying
$S_{12}$		1.5	I	A	Slow gallbladder emptying
$S_{13}$	*B*	600	I	A	Short gallbladder emptying duration
$S_{14}$		100	I	A	Long gallbladder emptying duration
$S_{15}$	$k_{12},\;k_{21}$	1.0, 0.1	II	D	Fast peripheral distribution with slow central return
$S_{16}$		0.1, 1.0	I	E	Slow peripheral distribution with fast central return
$S_{17}$	$v_{max}$	1.0	IV	A	Broad first pass elimination
$S_{18}$		0.02	I	A	Reduced first pass removal

^a^ Values of $k_{m}$ and $k_{ehc}$ are grouped in different cases represented by Roman numerals and capital letters, respectively; see Table 3.

**Table 3:** Values of the constants $k_{m}$ and $k_{ehc}$ and their effect on the percentage of drugs that undergo EHC.

*k_m_*				Effect on *EHC*% ^a^	*k_ehc_*					*EHC_max_*%
I	II	III	IV		A	B	C	D	E	
10	1	100	100	+	0.0666	0.3333	0.0133	0.7333	0.1333	20%
0.1	0.01	1	10	++	0.15	0.75	0.03	1.65	0.3	60%
0.001	0.0001	0.01	1	+++	0.4	2.0	0.08	4.4	0.8	80%

^a^ The effect is observed as a decrease with respect to the maximum value: insignificant decrease: +; moderate decrease: ++; outstanding decrease: +++.

To increase the diversity of the data generated in each scenario, the values of the parameters $k_{m}$ and $k_{ehc}$, which are related to the magnitude of the EHC, were varied. Table 3 shows the values of $k_{m}$ and $k_{ehc}$ for each of the cases and their effect on the EHC. Different combinations of these two parameters allowed the generation of up to nine different simulations per scenario, giving a total of one hundred and sixty-two simulations. At the end of each simulation, post-simulation calculations were performed to obtain the $AUC_{0-\infty }$.

From each simulation, time versus plasma concentration data were extracted at specific times to represent the sampling process. The sampling schemes were divided into three categories, which are shown in Table 4.

### 2.4. Empirical Pharmacokinetic Models

The empirical models were fitted to the data extracted from each simulation. Five models (M1 to M5) represented by functions were built to characterize the multiple peaks present in the EHC. They were compared with each other and with respect to the reference model of extravascular administration (M0), Equation (7). Equations (8) and (9) are modifications of previously reported models [37,38], while the rest, Equations (10)–(12), are new models built intuitively. They are described as:

(7) $$\begin{array}{c} C_{M0}\left(t\right)=a\left(e^{-bt}-e^{-ct}\right) \end{array}$$

(8) $$\begin{array}{c} C_{M1}\left(t\right)=at^{c}e^{-bt}+\overset{n}{\underset{i=1}{\sum }}\left(d_{i}e^{-{\left(\frac{t-f_{i}}{2g_{i}}\right)}^{2}}\right) \end{array}$$

(9) $$\begin{array}{c} C_{M2}\left(t\right)=e^{-at}\overset{n}{\underset{i=1}{\sum }}\left(\frac{b_{i}}{\Gamma \left(d_{i}\right)}\int_{0}^{tc_{i}}x^{d_{i}-1}e^{-x}dx\right) \end{array}$$

(10) $$\begin{array}{c} C_{M3}\left(t\right)=te^{-at}\overset{n}{\underset{i=1}{\sum }}\left(b_{i}\left\{\begin{array}{cc} 1-e^{-\frac{\left(t-c_{i}\right)}{d_{i}}}, & t-c_{i}\geq 0\\ 0, & t-c_{i}<0 \end{array}\right.\right) \end{array}$$

(11) $$\begin{array}{c} C_{M4}\left(t\right)=e^{-ct}\overset{n}{\underset{i=1}{\sum }}\left(b_{i}\left\{\begin{array}{cc} \frac{e^{2c\left(t-d_{i}\right)}-1}{e^{2c\left(t-d_{i}\right)}+1}, & t-d_{i}\geq 0\\ 0, & t-d_{i}<0 \end{array}\right.\right) \end{array}$$

(12) $$\begin{array}{c} C_{M5}\left(t\right)=C_{M4}\left(t\right)+e^{-ct}\overset{\infty }{\underset{j=1}{\sum }}\overset{n}{\underset{i=1}{\sum }}\left(e^{c24j-\frac{\left(24j+d_{i}-d_{1}\right)\left(\ln\left(\frac{b_{n}}{b_{1}}\right)+c\left(d_{1}-d_{n}\right)\right)}{d_{1}-d_{n}}}b_{i}\left\{\begin{array}{cc} \frac{e^{2c\left(t-d_{i}-24j\right)}-1}{e^{2c\left(t-d_{i}-24j\right)}+1}, & t-d_{i}-24j\geq 0\\ 0, & t-d_{i}-24j<0 \end{array}\right.\right) \end{array}$$

where the letters a, b, c, d, etc., are the parameters of the models; t is the time and $C_{M}\left(t\right)$ the plasma concentration estimated by each model. Except for the model given by Equation (1), the models are built with summations that describe the first absorption peak (when i=1) and secondary resorption peaks caused by EHC (when i>1); therefore, in these models, the number of peaks is equal to n. Another characteristic that all models have in common is that they incorporate a term or factor to the left of the sum and that characterizes the elimination process.

The model described by Equation (12) is an extension of the model in Equation (11) and is intended to predict the presence of additional peaks that may not be detected after the first 24 h. This prediction is made under the assumption that the gallbladder will empty with an intensity and at a time like the first day. In this model, the intensity and moment of gallbladder emptying are given by the parameters $b_{i}$ and $d_{i}$, respectively. The prediction after 24 h is based on the parameters that describe the largest peak (usually the first absorption peak, $b_{i}$ and $d_{i}$) and the last observed peak ($b_{n}$ and $d_{n}$).

The $AUC_{0-\infty }$ of all models converge to a finite value when the model is fitted to time versus concentration data; its value can be calculated exactly using explicit formulas. Equations (S1)–(S6) shown in the Supplementary Materials (Supplementary Data S1) allow us to perform this calculation exactly, and they were obtained with the help of Maple software version 2018.

### 2.5. Compilation of Pharmacokinetic Profiles from the Literature

A bibliographic search and compilation of pharmacokinetic profiles of drugs that undergo EHC reported in different works was carried out. The search criteria were drugs administered orally, in a single dose, and with a profile with at least two clearly visible peaks. WebPlotDigitizer version 4.5 software was used to recover time and plasma concentration data from the graph reported in the study. The software allows you to select the points in the pharmacokinetic profile to estimate the values of the time–concentration data pairs. This software has been used successfully for data collection from graphs [43].

### 2.6. Data Fitting, Statistical Analysis, and Evaluation of Empirical Models

Non-linear regression (NLR) of the simulated data retrieved from the literature was performed using the Curve Fitting tool of MATLAB version R2021a. When necessary, restrictions were applied to the coefficients to obtain a good parametric fit. The adjusted $R^{2}$, SSE, and RMSE error metrics were obtained directly from said tool. The script used to execute the NLR is provided in the Supplementary Materials (Supplementary Data S2).

Evaluation of models was carried out with the corrected Akaike Criterion (AICc) for small sample sizes using Equation (13):

(13) $$\begin{array}{c} AICc=n\ln\left(\frac{SSE}{n-k}\right)+2k+\frac{2k\left(k+1\right)}{n-k-1} \end{array}$$

where k is the number of model parameters, and n is the number of observations.

As part of evaluation of the models, comparisons were made between the reference $AUC_{0-\infty }$ of the simulation, with the $AUC_{0-\infty }$ calculated by the trapezoidal method and estimated by empirical models, Equations (S1)–(S6).

## 3. Results

### 3.1. Effect of EHC Degree on AUC

**Figure 2:**
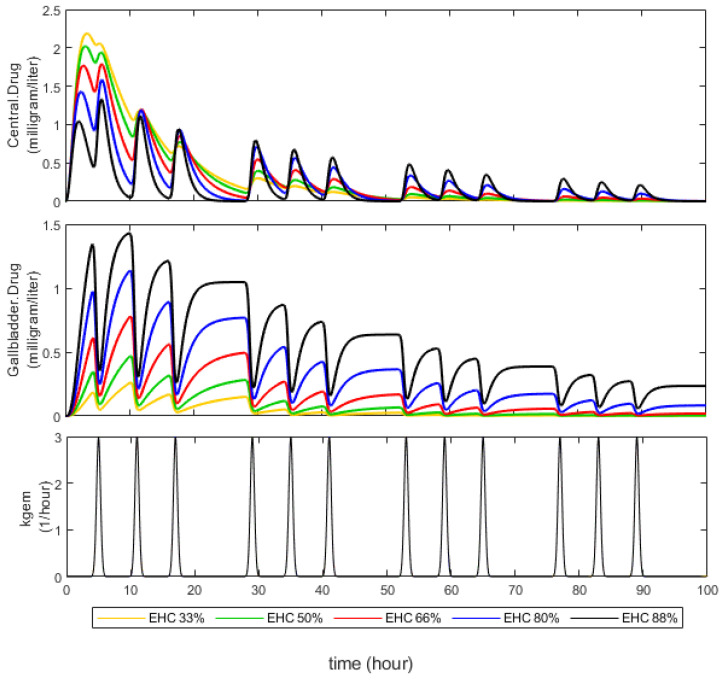
Pharmacokinetic simulations at different percentages of drugs undergoing enterohepatic circulation. The plasma profile in the middle of the drug concentrations in the gallbladder and below the kinetics of gallbladder emptying are shown above, expressed as variation in the *k_gem_* according to Equation (3).

Initially, we aimed to verify whether the compartmental model could accurately simulate the EHC process, allowing us to investigate its impact on AUC. Simulations following single-dose extravascular administration delineated the relationship between gallbladder emptying, gallbladder drug concentrations, and plasma profile (Figure 2). Notably, spikes in plasma profile coincided with gallbladder contractions, underscoring the dynamic nature of EHC. Additionally, drugs subject to extensive EHC (depicted by the black line) exhibited prolonged residence in the organism compared to those experiencing minimal EHC (illustrated by the yellow line).

**Figure 3:**
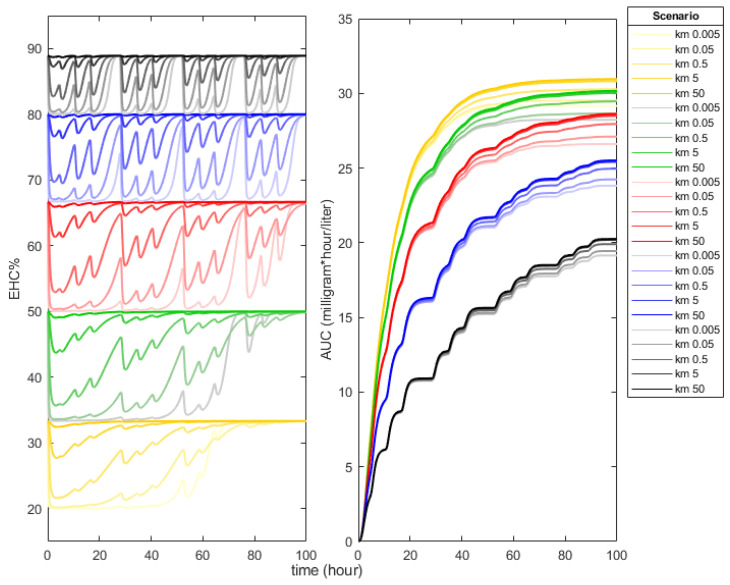
Effect of *EHC*% on cumulative AUC. On the (**left**), the temporal dynamics of the percentage of drugs subjected to *EHC*% is shown, and on the (**right**), the temporal increase in the cumulative *AUC* is shown.

Expanding our model to encompass biotransformation and gallbladder transport enabled us to assess the influence of their interplay. Simulations with a $k_{m}=50$ revealed a consistent EHC% value (bold lines, left panel of Figure 3), suggesting negligible competition. Conversely, simulations with $k_{m}=0.005$ yielded fluctuating EHC% values, ranging between a minimum and a theoretical maximum determined by Equation (6) (pale lines, right panel of Figure 3), indicative of significant competition. In this scenario, maximum EHC% values ranged from 88% to 33%. This competitive dynamic directly impacted AUC, where cases of negligible competition yielded higher maximum values (ranging from 20.2 to 30.9 mg × h/L) compared to scenarios with relevant competition, where AUC decreased with decreasing $k_{m}$ values. These findings affirm the completeness and realistic simulation capabilities of our model to simulate the EHC process.

### 3.2. Simulation of Pharmacokinetic Scenarios and Fitting to Empirical Models

We conducted 162 simulations across eighteen scenarios to explore their impact on $AUC_{0-\infty }$, as summarized in Table 5. Comparative analysis revealed that scenarios S1 through $S_{6}$ and $S_{15}$ exhibited significantly different $AUC_{0-\infty }$ compared to standard model $S_{7}$. Additional details and pharmacokinetic simulation plots for each scenario are available in the Supplementary Materials (Supplementary Data S3, Figures S1–S18).

**Figure 4:**
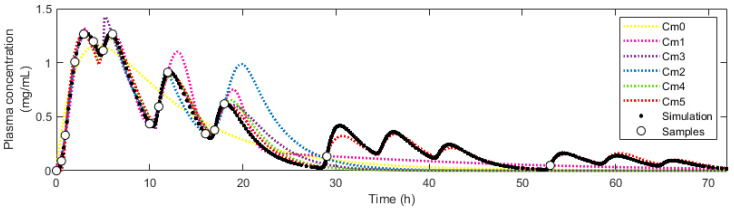
Fitting of empirical models to data from a representative simulated pharmacokinetic profile. In this figure, the adjustment was made to the data obtained through a meal-based sampling, where up to 80% of the drug undergoes EHC.

Next, we fitted six empirical models to the obtained plasma profile data. Figure 4 illustrates the fit of these models to the data from a representative simulation. Apart from model M0, all models successfully captured the multiple peaks of the plasma profile, exhibiting subtle visual differences.

**Figure 5:**
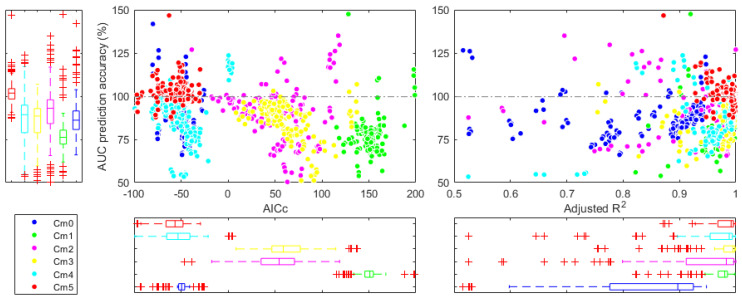
Scatter plots of the accuracy of *AUC* versus *AICc* and *R*^2^ of each model (*M*0–*M*5).

Criteria for selecting the best model included adjusted $R^{2}$, AICc, and accuracy in predicting AUC. Figure 5 displays scatter plots of these criteria, while Table 6 presents their means for each model. Figure 5 allows the visual identification of the result of each regression of the empirical models in a scatter plot. Models M4 (cyan balls) and M5 (red balls) stand out for showing a higher degree of clustering than other models, and both models have the lowest AICc values and the highest adjusted $R^{2}$ values. The gamma distribution model (pink balls) presented poorer clustering due to the greater difficulty of performing reproducible regressions since, in this model, applying restrictions for a successful NLR was less intuitive for the authors.

Model M5 achieved the highest values for adjusted $R^{2}$ (0.979), AICc (−58.1), and accuracy in predicting AUC (105.9). Although M5 exhibited the smallest spread in adjusted $R^{2}$ (±0.02), it also had the highest spread in AUC prediction accuracy (±21.4). These results indicate that M5 demonstrated the best performance characteristics and was thus chosen for subsequent analysis.

### 3.3. Estimation of AUC in Different Pharmacokinetic Scenarios

**Figure 6:**
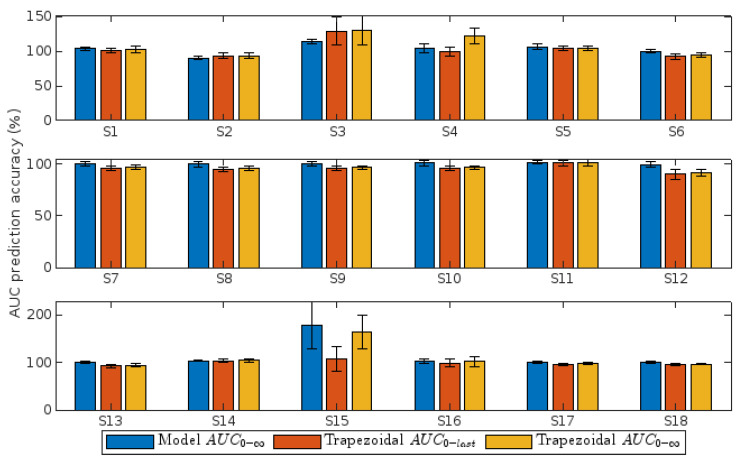
Accuracy of *AUC* predicted by model *M*5 and by the trapezoidal method for each pharmacokinetic scenario. Calculation was made from data obtained from a meal-based sampling.

Applicability of model M5 to various pharmacokinetic scenarios was examined. Results indicated that model M5 accurately predicted AUC for most scenarios, with close to 100% accuracy (Figure 6). However, scenarios $S_{3}$ (rapid renal elimination from central compartment) and $S_{15}$ (fast peripheral distribution with slow central return) notably overestimated the actual AUC (exceeding 110% and 150%, respectively). This overestimation persisted regardless of whether the trapezoidal method or model M5 was employed.

### 3.4. Estimation of AUC under Different Sampling Schemes

**Figure 7:**
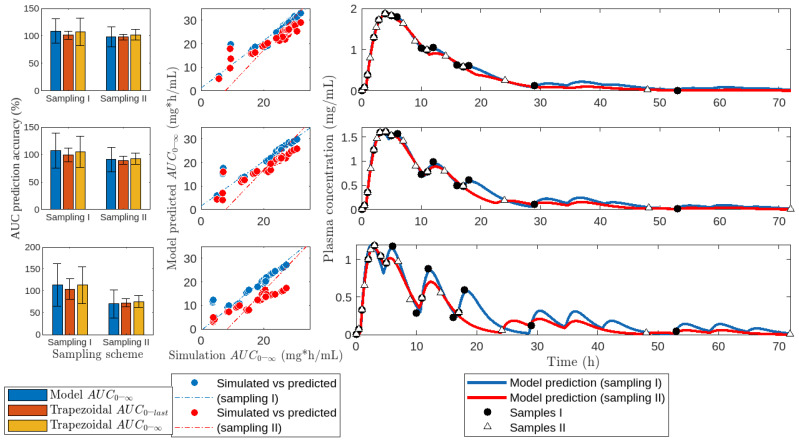
Fit of model *M*5 to drug profiles submitted to 40% (**top**), 60% (**middle**), and 80% (**bottom**) of *EHC*%. Data were adjusted using meal-based (Sampling I) and conventional (Sampling II) samplings, as shown in Table 4.

Next, we investigated the impact of sampling schemes on predictions of model M5. Firstly, we contrasted meal-based and conventional sampling methods (Figure 7). Notably, when the percentage of drugs undergoing EHC was 40%, model-predicted AUC accuracy was nearly 100% for both sampling schemes. However, at 80% EHC, accuracy decreased to approximately 50% with conventional sampling, while remaining close to 100% with meal-based sampling. Regardless of the sampling scheme or the percentage of drugs undergoing EHC, AUC estimates by the trapezoidal method closely mirrored those by the model.

**Figure 8:**
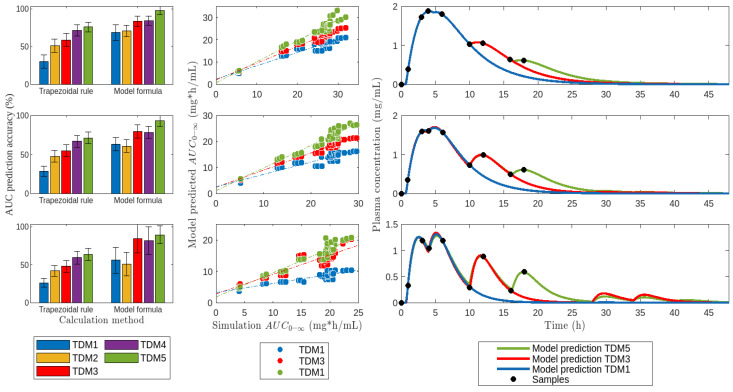
Fit of model *M*5 to drug profiles submitted to 40% (**top**), 60% (**middle**), and 80% (**bottom**) of *EHC*%. Adjustment to the data was performed by sampling for the TDM, as shown in Table 4.

Secondly, different sampling schemes for TDM were assessed (Figure 8). It was observed that with TDM1 and TDM2 schemes, model-predicted AUC accuracy ranged between 50–75%, depending on the percentage of drugs undergoing EHC. In contrast, TDM3 and TDM4 schemes notably improved accuracy to over 75%, while TDM5 showed a modest increase. Notably, AUC predictions by the model were nearly identical with TDM1/TDM2 and TDM3/TDM4 schemes, respectively. In all cases, AUC estimates by the trapezoidal method improved gradually with increased sampling times.

### 3.5. Application of Model M5

Fourteen average plasma profiles of drugs undergoing EHC were gathered from the literature [44,45,46,47,48,49,50,51,52,53,54,55,56], and model M5 was applied to fit these profiles. Additional graphs provided in the Supplementary Materials illustrate the fit of model M5 to data extracted from representative pharmacokinetic profiles (Supplementary Data S4, Figures S19–S28).

**Figure 9:**
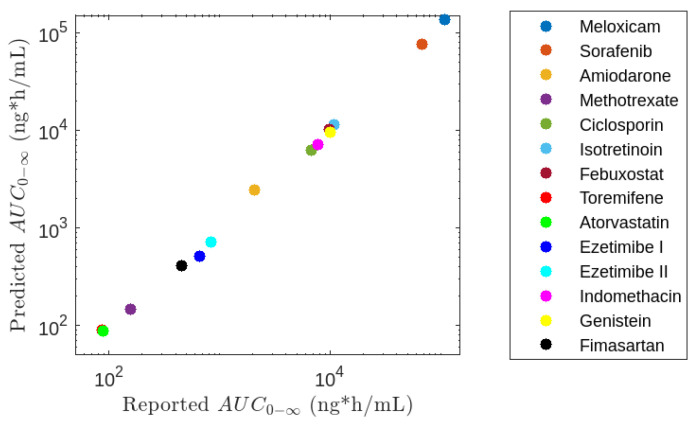
Comparison between reported for drugs that undergo EHC and *AUC* predicted by model *M*5.

The AUC was estimated using model M5 and compared with reported values, as shown in Figure 9, obtaining a correspondence between both values close to 100%. The largest discrepancies were observed for Ezetimibe and Meloxicam, with differences of 78.4% and 124.1%, respectively, while other estimates fell within the range of 80–120%, as depicted in Table 7.

## 4. Discussion

### 4.1. Effect of EHC Degree on AUC

Simulations of our compartmental model are in agreement with other studies in which it is stated that the EHC increases the permanence of the drug in the body by increasing $t_{1/2}$ apparent elimination [40,57]. We want to highlight the linear nature of our compartmental model in which an increase in dose proportionally increases AUC regardless of the degree of EHC% (Supplementary Data S5, Figure S29). This is not surprising considering that drugs such as atorvastatin and mycophenolic acid that experience EHC maintain linear pharmacokinetics with respect to AUC [58,59]. However, a less understood effect of the EHC is its impact on AUC. We take advantage of the information generated from the simulations to address a question that has been discussed in other works.

It has been discussed on several occasions how AUC is altered when the drug undergoes EHC. The prevailing position is that AUC increases when EHC exists; several papers have provided evidence to support this position [60,61,62,63]. However, in other studies, it has been proposed that the degree of EHC does not have an impact on AUC [57,64]. Recently, Ibarra et al. reported that the inclusion of a hepatic compartment, where the competition between hepatobiliary secretion and biotransformation is explicit, allows us to explain within their model why the level of AUC increases when the degree of EHC is higher [40]. Similar to the model proposed by Ibarra, in our model, this competition is considered with the difference that the elimination constant by biotransformation is not of the first order but follows Michaelis–Menten kinetics.

For drugs with a very large $k_{m}$, the rate of elimination by biotransformation ($v_{bio}$) is negligible, but when the $k_{m}$ rate is very small, $v_{bio}$ increases, and the competition between bile secretion and biotransformation becomes important. When this happens, $v_{max}$ is reached more quickly due to the drug’s high affinity for the enzyme. As described above, the competition between the two processes causes a fluctuation of EHC% between a theoretical minimum and a theoretical maximum. The maximum can be estimated with Equation (6), while Equation (14) allows estimating the minimum possible for EHC%, which is reached if the concentration of drug in the compartment is high enough.

(14) $$\begin{array}{c} \begin{array}{c} EHC_{min}\%=\frac{k_{ehc}}{k_{ehc}+k_{rel}+k_{12}+v_{max}}\times 100\% \end{array} \end{array}$$

Figure 3 shows that AUC of drug is lower at $EHC_{max}\%$ equal to 88% compared to those with $EHC_{max}\%$ equal to 33%. Within the proposed model, this is explained by the fact that biliary transport behaves as an additional elimination pathway that results in a decrease in $AUC_{0-\infty }$ by the amount of the drug extracted from the central compartment.

According to our model, we can differentiate two ways that increase AUC by altering the degree of EHC that a drug undergoes. The first is by decreasing $k_{m}$ of biotransforming enzyme by adding a competitor, and the second is by suppressing drug transport into the gallbladder by decreasing the value of $k_{ehc}$. We translate these two possibilities into a couple of examples.

In the first case, it could happen that a drug that is subjected to EHC and to biotransformation is administered concomitantly with another drug that is also biotransformed by the same enzyme (e.g., CYP3A4). Competition of both substrates for the same enzyme will increase $k_{m}\;$ of the drug that EHC is experiencing. As shown in Figure 3, high values of $k_{m}\;$ increase AUC. In fact, simvastatin, a drug that undergoes EHC, and cyclosporine are biotransformed by the same enzyme CYP3A4; when administered concomitantly, the body’s exposure to simvastatin increases enough to give rise to the toxic effects of simvastatin [65].

In the second case, a drug could be presented that undergoes EHC in the normal population, but where a mutation in the gene encoding a protein responsible for drug transport to the biliary canniculus (e.g., MRP2) is able to decrease the activity of the transporter in carriers of the mutation. According to our model, this mutation would imply a decrease in $EHC_{max}\%$ and an increase in AUC with respect to the normal population. In fact, people who suffer from the so-called Dubin–Johnson syndrome have higher levels of bilirubin in plasma than a normal person because the MRP2 transporter is defective, preventing bilirubin from experiencing enterohepatic circulation to which it is normally subjected, which causes its accumulation within the body [66].

In summary, suppressing biliary transport or favoring biliary transport over biotransformation could increase AUC of drugs from two different perspectives. In one study, both perspectives were investigated and reached conclusions similar to those of our model [67], but more studies are still needed to investigate how much of the AUC increase observed in these cases can be attributed to the modification of the EHC.

### 4.2. Simulation of Pharmacokinetic Scenarios and Fit to Empirical Models

In addition to the biliary transfer rate ($k_{ehc}$), we can point out from Table 5 that the parameters related to absorption ($k_{a})$, renal elimination ($k_{rel})$, intestinal elimination ($k_{gel})$, and transport between compartments ($k_{12}$ y $k_{21})$ were the ones that most influenced the modification of $AUC_{0-\infty }$ with respect to the standard model. Regardless of the effect that these processes may have, all of them have the characteristic pharmacokinetic profile of “sawtooth”, a sufficient requirement for their adjustment to empirical models.

A qualitative analysis of Figure 4 reveals that except for the classical model of extravascular administration (M0), most models can capture the multi-peak phenomenon, although with some differences. For example, the model M1 generated symmetric peaks, while the rest of the models generated asymmetric peaks more similar to those in the simulation. The models M1 and M2 presented a smooth transition from one peak to the next, while in the rest of these models, the transition was more abrupt. The models M1 and M2 tended to overestimate the magnitude of some of the peaks. The models M4 and M5 generated nearly identical predictions in the first 24 h, but M5 stood out for the ability to predict subsequent peaks even if they were not identified by the sampling method.

On the other hand, a quantitative analysis of the results in Table 6 shows that increasing the number of model parameters does not necessarily imply an adjusted $R^{2}$ value closer to 1. Models that required more parameters (M1, M2, and M3) had lower $R^{2}$ values than models that required fewer parameters (M4 and M5). However, the number of parameters increases if you increase the value of AICc. In fact, model M1, which requires up to 14 parameters to fit a profile with four peaks, had the highest AICc value of all the models evaluated. As complex as it may seem, model M5, described by Equation (12), requires relatively few parameters to describe various peaks, which is reflected in the smallest AICc of all the models evaluated.

It is important to mention that the greater the number of secondary peaks that you want to model, the greater the number of parameters. Increasing the number of parameters also implies increasing the number of samples; otherwise, the NLR could not be performed. The number of samples must be greater than or equal to the number of parameters to be used to characterize one or more peaks. Table 8 shows the number of parameters (and, therefore, minimum samples required) to characterize the peaks of a plasma profile with EHC. Depending on the number of peaks observed, you might choose to use more or fewer parameters for a given model.

Regarding the accuracy of the prediction AUC, it is interesting to note that even the classical model of extravascular administration (M0) that is unable to capture secondary peaks has an accuracy in AUC estimation similar to some of the models that can capture secondary peaks. Previously, we presented an empirical model to capture the secondary peaks of the plasma profile of simvastatin [38], which is equivalent to the model M1 of this work. Unlike our previous work, this time, the biexponential model M0 was better at estimating AUC than the model M1. This suggests that model M1 might fit well with the plasma profiles of simvastatin but not with other drugs with different pharmacokinetic characteristics.

With respect to the model first proposed by Premaud et al. [37] and that in this work, which is equivalent to the model M2, a couple of questions can be said. This model has undoubtedly been the most successful in practice, as it has demonstrated its superiority in the field of TDM over the fixed dose in the monitoring of mycophenonlate mofetil, a drug with a narrow therapeutic margin [68]. Adaptations to it have even been proposed for the case of profiles with three peaks, which has allowed its usefulness to be further expanded [39]. In this paper, we present the model in its general form to be able to model any number of peaks, Equation (9), and we also provide a formula for the exact calculation of $AUC_{0-\infty }$ from its parameters, Equation (S3). However, in this work, there were models that presented a better performance; this was mainly because other models required a smaller number of parameters to model the same pharmacokinetic profile. In addition, on many occasions, the execution of the NLR was less intuitive for M2 compared to other models such as M3, M4, and M5.

### 4.3. Estimation of AUC in Different Pharmacokinetic Scenarios

Once the superiority of model M5 had been demonstrated, we proceeded to evaluate under which conditions it could be more useful and in which it does not offer significant improvements compared to the trapezoidal method. As mentioned above, the M5 model overestimated AUC in scenarios $S_{3}$ and $S_{15}$. This suggests that AUC predictions from model M5 are more likely to be inaccurate for drugs that are eliminated very quickly or those with two-compartment kinetics in which the drug remains for longer in the body. It is important to note that kinetic equilibrium is not reached instantaneously between compartments, and the addition of a peripheral compartment in scenario $S_{15}$ makes it possible to take into account the presence of highly lipophilic drugs. Considering this, model M5 might have trouble estimating AUC of highly lipophilic drugs.

An important aspect to highlight is that AUC calculation for each scenario using Equation (S6) was not significantly different from the one calculated by the trapezoidal method. These results suggest that, if possible, the selection of a meal-based sampling scheme is more important than the selection of a complex model that fits the data well but does not offer practical improvements beyond a good fit.

In the past, it has been argued that possibly the use of the trapezoidal method in drugs undergoing EHC could lead to an overestimation of AUC. This assumption is based on the fact that the lack of resolution of the secondary peaks would lead to an overestimation of the actual area of these peaks, similar to approximating the area of a circle inscribed within a square from the area of that square. However, in this paper, we observed that, more often than not, the trapezoidal method estimates the real quite well, at least when there is an exhaustive sampling based on meals; see Figure 6.

### 4.4. Estimation of AUC under Different Sampling Schemes

The results suggest that it is more important to opt for a meal-based sampling method when secondary peaks are very pronounced due to a high degree of EHC than when peaks are very small due to a low degree of EHC. Even so, in practice, it would be worthwhile to opt for meal-based sampling whenever possible, as the contribution of the EHC process to the AUC of some drugs can be highly variable. For example, the variability of EHC in mycophenolic acid ranges from 10 to 60% [69].

It is important to note that in a comprehensive meal-based or conventional sampling, the AUC calculated by the trapezoidal method is very similar to that calculated by model M5; see Figure 7. This result suggests that when sufficiently large numbers of samples are available, as in the case of bioequivalence studies, the differences between each of the methods for calculating AUC are negligible. In these cases, opting for the trapezoidal method would be the most practical.

When sampling was limited, we found that increasing the number of samples does not necessarily lead to an improvement in the estimation of AUC. True improvements in the estimation of AUC were observed between TDM2 to TDM3 and TDM4 to TDM5 schemes. A more in-depth analysis of Figure 8 shows that the TDM1 and TDM2 samples allow the characterization of only two peaks, TDM3 and TDM4 three peaks and TDM5 four peaks of the plasma profile. The clear conclusion is that it is more important to increase the number of properly characterized peaks than to increase the number of samples.

It is important to note that in our study, the sampling times for TDM were not chosen arbitrarily but followed the rationality of meal-based sampling. From Figure 8, it is easy to see that the sampling times coincide almost exactly with the maximums and minimums of the plasma profile. These results suggest that in the TDM of drugs subject to EHC, it is essential to establish a meal schedule for the planning of sampling and that these are taken for a sufficiently long time to characterize several peaks. In this sense, our work supports the suggestions made by Lixuan et al., who studied the effect of mealtimes on AUC of mycophenolic acid [70].

### 4.5. Application of Model M5

Our study has the limitation that it does not have data from individual real-world profiles; we only had access to the average plasma profiles of some drugs published in other studies. Even so, we were able to adjust model M5 to all the profiles collected and obtained, in most cases, the estimates of AUC that were quite close to those reported in their respective articles. In the process, we obtained profiles with dynamics for which we cannot be sure that they approximate the real dynamics since several of these studies did not report or consider the mealtimes for sampling. The adjustment of the proposed model to these profiles is the first step to promote its application in data generated from real-world studies based on rational sampling.

It is clear that one of the deficiencies of model M5 is its inability to estimate the degree of EHC; however, as seen in Table 7, this model estimates the AUC quite well. It is not easy to estimate experimentally the degree of EHC that a drug experiences, and there are few works that report this value. Due to the inability to perform an analysis of the fit of model M5 to the profiles of the drugs studied from these data, we opted to use the elimination half-life $t_{1/2}$ to perform this analysis.

We previously pointed out that the M5 model had difficulties in adequately estimating the AUC in those scenarios that considered drugs with very long permanence in the body. In Table 7, we find precisely that model M5 considerably overestimated the AUC of meloxicam and amiodarone, which have a very high $t_{1/2}$ (39.5 h and more than 25 days, respectively). At the other extreme, model M5 underestimated the AUC of ezetimibe although it does not have a particularly high $t_{1/2}$ (between 13.8 and 15.1 h).

It should be clarified that although the empirical models were applied to data generated from simulations of the EHC process, in theory, they could be applied to any other profile that has multiple peaks caused by mechanisms other than the EHC. This would be possible since, being of an empirical nature, they do not need the determination of kinetic parameters of physiological or physicochemical basis. In this way, they could be applied to plasma profiles that show multiple peaks, such as those caused by pulsatile delivery systems [71,72] (examples of these commercially available pulsatile systems are Pulsincap^®^, Ritalin^®^, Pulsys^®^) or those caused by physicochemical differences between different regions of the gastrointestinal tract. It has been suggested that differences in the pH of the gastrointestinal tract that ionize mycophenolic acid and not EHC could be responsible for the double-peak phenomenon observed in this drug [73]. However, it would be inapplicable to vascular delivery drug profiles that exhibit multiple spikes regardless of the mechanism behind these spikes (e.g., fentanyl, sufentanil, propofol, and phenoperidine [74,75,76,77]).

We highlight the feasibility of applying model M5 as a structural model in population pharmacokinetic models. Many structural models employed in population pharmacokinetic approaches have used physiological or mechanism-based concepts to describe the EHC [78]. An example of the success of this type of model contains forty-five structural parameters and simultaneously describes the extent and time course of EHC in rats, dogs, and humans for fimasartan [79]. However, empirical models with fewer parameters might present advantages as structural models. The most recent example is the non-linear mixed effect (NLME) approach based on stochastic approximation expectation–maximization for the double gamma distribution model [80].

Our model is unable to predict the effect on the pharmacokinetic profile of variables such as age, gender, body weight, pathological states, etc. In this sense, the inclusion of covariates in our model for the development of further population pharmacokinetic models would allow us to limit its application to specific clinical environments in the same way that has been done for the model proposed by Premaud et al. [37].

## 5. Conclusions

In this study, we conducted simulations to assess the impact of EHC on AUC in various scenarios. Our findings indicate that modulating biliary transport can effectively increase AUC. We evaluated five empirical models for characterizing EHC peaks, identifying model M5 as the most efficient due to its simplicity, accurate AUC estimates, and ability to predict peaks beyond sampling times. While model M5 performed well in most scenarios, it struggled with drugs exhibiting rapid renal elimination or bicompartmental kinetics.

Evaluation of different sampling schemes revealed that our proposed model did not consistently outperform the trapezoidal method, particularly with extensive sampling. However, limited sampling for TDM benefited from our model’s ability to characterize peaks, yielding superior AUC estimates compared to the trapezoidal method. In conclusion, this study underscored the importance of implementing meal-based sampling schemes and laid the groundwork for applying empirical models in real-world studies of drugs undergoing EHC.

## Figures and Tables

**Table 4:** Sampling schemes.

Sampling Type		Sampling Times (h)	Number of Samples	Description
Meal-based sampling		0, 0.5, 1, 2, 3, 4, 5, 6, 10, 11, 12, 16, 17, 18, 29, 53, 77	17	Focuses on sampling before and after food intake
Conventional sampling		0, 0.25, 0.5, 0.75, 1, 1.5, 2, 2.5, 3, 4, 5, 7, 9, 12, 24, 48, 72	17	Prioritize sampling at the beginning to characterize $t_{max}$$\;\mathrm{and}\;C_{max}$
Sampling for TDM	TDM1	0, 1, 3, 4, 6	5	Reduce the number of samples and restrict their taking to the first hours after administration
	TDM2	0, 1, 3, 4, 6, 10	6	
	TDM3	0, 1, 3, 4, 6, 10, 12	7	
	TDM4	0, 1, 3, 4, 6, 10, 12, 16	8	
	TDM5	0, 1, 3, 4, 6, 10, 12, 16, 18	9	

**Table 5:** Average $AUC_{0-\infty }$ per scenario.

Scenario	$AUC_{0-\infty }$	Scenario	$AUC_{0-\infty }$	Scenario	$AUC_{0-\infty }$
$S_{1}$ ^b^	26.45 ± 2.73	$S_{7}$	23.91 ± 3.24	$S_{13}$	23.77 ± 3.31
$S_{2}$ ^b^	16.51 ± 3.86	$S_{8}$	23.86 ± 3.25	$S_{14}$	24.10 ± 3.13
$S_{3}$ ^b^	4.89 ± 0.64	$S_{9}$	23.89 ± 3.24	$S_{15}$ ^a,b^	9.74 ± 4.34
$S_{4}$ ^a,b^	87.52 ± 9.84	$S_{10}$	23.89 ± 3.23	$S_{16}$	20.54 ± 4.54
$S_{5}$ ^b^	12.86 ± 3.56	$S_{11}$	24.07 ± 3.15	$S_{17}$	23.49 ± 3.38
$S_{6}$ ^b^	29.06 ± 2.09	$S_{12}$	23.38 ± 3.51	$S_{18}$	24.64 ± 3.25

^a^ Due to the greater persistence of drugs in the organism, simulation time was longer than 96 h in these scenarios. ^b^ Scenarios with *p*-values less than 0.05.

**Table 6:** Means of the criteria for model selection.

Model	$\mathrm{Adjusted}\;R^{2}$	AICc	Accuracy (%) $AUC_{0-\infty }$
*M*0	0.827 ± 0.14	−51.9 ± 12.3	88.4 ± 18.7
*M*1	0.940 ± 0.17	150.4 ± 12.6	75.9 ± 13.0
*M*2	0.888 ± 0.26	52.1 ± 31.8	89.6 ± 17.3
*M*3	0.931 ± 0.20	63.3 ± 25.8	84.2 ± 14.0
*M*4	0.943 ± 0.12	−51.1 ± 19.1	86.4 ± 16.2
*M*5	0.979 ± 0.02	−58.1 ± 14.7	105.9 ± 21.4

**Table 7:** Results of NLR and *AUC* estimation of representative drugs.

Drug	Number of Samples	Number of Parameters	Accuracy (%) $AUC_{0-\infty }$	$\mathrm{Adjusted}\;R^{2}$	$\mathrm{Half}\text{-}\mathrm{Life}\;t_{1/2}$ (h)	Reference
Meloxicam	11	9	124.1	0.8013	39.5	[44]
Sorafenib	14	9	110.6	0.8791	25.3	[45]
Amiodarone	15	9	117.6	0.9689	5.1 ^a^	[46]
Methotrexate	12	9	95.4	0.9684	5.3	[47]
Cyclosporin	11	9	93.6	0.9494	8.1	[48]
Isotretinoin	22	9	104.6	0.9591	23.5	[49]
Febuxostat	19	9	102.1	0.9652	2.78	[50]
Toremifene	10	9	104.1	0.9912	29.7	[51]
Atorvastatin	17	9	98.5	0.9400	3.9	[52]
Ezetimibe	25	9	78.4	0.8524	15.1	[53]
Ezetimibe	25	9	84.1	0.8586	13.8	[53]
Indomethacin	23	9	92.2	0.9736	6.8	[54]
Genistein	11	9	95.1	0.9527	4.1	[55]
Fimasartan	17	9	90.5	0.7552	5.6	[56]

^a^ The elimination half-life of amiodarone is biphasic; the half-life of the first phase of disposition is presented here since the actual half-life is between 25 and 110 days.

**Table 8:** Number of parameters required to characterize a plasma profile.

Model	Minimum Number of Parameters and Samples per Peak ^a^			
	1	2	3	4
*M*0	3	3	3	3
*M*1	5	8	11	14
*M*2	4	7	10	13
*M*3	4	7	10	13
*M*4	3	5	7	9
*M*5	3	5	7	9

^a^ Refers to the minimum number of parameters required by each model to characterize 1, 2, 3, and 4 peaks of the plasma profile.

## Data Availability

The data presented in this study are available upon request from the corresponding author.

## Supplementary Materials

The following supporting information can be downloaded at: https://www.mdpi.com/article/10.3390/pharmaceutics16081044/s1, Supplementary Data S1: Equations for AUC calculation; Supplementary Data S2: Source script; Supplementary Data S3: Simulations of pharmacokinetic scenarios; Supplementary Data S4: Fit of model M5 to selected plasma profiles; Supplementary Data S5: Linearity of compartmental model.
