# Supplementary material for: Pharmacokinetic Simulation and Area under the Curve Estimation of Drugs Subject to Enterohepatic Circulation
Source: Pharmaceutics. 2024 Aug 6;16(8):1044. doi: 10.3390/pharmaceutics16081044 (PMC11360071; doi:10.3390/pharmaceutics16081044)
Supplement: Supplementary file 1 [file pharmaceutics-16-01044-s001.zip › pharmaceutics-3071328-supplementary.pdf]

Pharmacokinetic Simulation and AUC Estimation of Drugs Subject to Enterohepatic Circulation
(Supplementary Materials)

Supplementary Data S1: Equations for AUC calculation.

$$AUC_{0-\infty_{M0}} = \frac{a}{c} - \frac{a}{b} \tag{S1}$$

$$AUC_{0-\infty_{M1}} = \frac{a\Gamma(c+1)}{b^{c+1}} + \frac{\sqrt{\pi}}{2}\sum_{i=1}^{n} d_i g_i + \frac{\sqrt{\pi}}{2}\sum_{i=1}^{n} f_i g_i \mathrm{erf}\left(\frac{f_i}{g_i}\right) \tag{S2}$$

$$AUC_{0-\infty_{M2}} = \frac{1}{a}\sum_{i=1}^{n} b_i \left(\frac{c_i}{c_i + a}\right)^{d_i} \tag{S3}$$

$$AUC_{0-\infty_{M3}} = \frac{1}{a^2}\sum_{i=1}^{n} \frac{b_i e^{-\tau_i a}(1 + c_i a + 2d_i a + c_i d_i a^2)}{(d_i a + 1)^2} \tag{S4}$$

$$AUC_{0-\infty_{M4}} = \frac{\pi - 2}{2c}\sum_{i=1}^{n} b_i e^{-d_i c} \tag{S5}$$

$$AUC_{0-\infty_{M5}} = \frac{\pi - 2}{2c}\left(\sum_{i=1}^{n} b_i e^{-d_i c} + a\sum_{i=1}^{n} b_i \left(\frac{b_n}{b_1}\right)^{-\frac{d_i}{d_1 - d_n}} e^{-2d_i c}\right), \quad a = \frac{\left(\frac{b_n}{b_1}\right)^{\frac{d_1}{d_1 - d_n}} e^{d_1 c}}{\left(\frac{b_n}{b_1}\right)^{\frac{24}{d_1 - d_n}} e^{24c} - 1} \tag{S6}$$

Supplementary Data S2: Source script.

Simulation data merge.

```
% This script concatenates the data from each simulation of each pharmacokinetic scenario
into the Cp (Plasma concentration) and Time matrices.
% S1.
for i = 1:9
    s(1,i) = height(s01(i, 1).Data);
    Cp(:,i) = vertcat(s01(i, 1).Data(:,1),zeros(1000-s(1,i),1));
    Time(:,i) = vertcat(s01(i, 1).Time(:,1),zeros(1000-s(1,i),1));
end
```

Sampling.

```
% Choose sampling times to extract drug concentration from each simulation.
for i = 1:162
    n = [0,0.5,1,2,3,4,5,6,10,11,12,16,17,18,29,53,77];
    [val,A] = min(abs(Time(:,i)-n));
    time(:,i) = Time(A,i);
    Drug(:,i) = Cp(A,i);
end
```

Initialization.

```
% Initialize arrays to store fits and goodness-of-fit.
fitresult = cell( 162, 1 );
```

```
gof = struct( 'sse', cell( 7, 1 ), ...
    'rsquare', [], 'dfe', [], 'adjrsquare', [], 'rmse', [] );
```

Fit: 'Model 5'.

```
% This script gives the instructions to carry out non-linear regression of model 5 for the
prediction of peaks at times greater than 24 hours, based on the first peak observed in the
profile.
for i = 1:162
    [xData, yData] = prepareCurveData( time(:,i), Drug(:,i) );

    % Set up fittype and options.
    ft = fittype('exp(-a*x)*((x<c).*0 + (x>=c).*(b*((exp(2*a*(x-c)))-1)/((exp(2*a*(x-
c)))+1))+(x<e).*0+(x>=e).*(d*((exp(2*a*(x-e)))-1)/((exp(2*a*(x-e)))+1))+
(x<g).*0+(x>=g).*(f*((exp(2*a*(x-g)))-1)/((exp(2*a*(x-
g)))+1))+(x<k).*0+(x>=k).*(h*((exp(2*a*(x-k)))-1)/((exp(2*a*(x-k)))+1))+(x<(e+24)).*0
+(x>=(e+24)).*(d*exp(-(log(h/b)-a*(k-c))*((24+e-c)/(c-k)))*exp(a*24)*((exp(2*a*(x-e-24)))-
1)/((exp(2*a*(x-e-24)))+1))+(x<(g+24)).*0 +(x>=(g+24)).*(f*exp(-(log(h/b)-a*(k-c))*((24+g-
c)/(c-k)))*exp(a*24)*((exp(2*a*(x-g-24)))-1)/((exp(2*a*(x-g-24)))+1))+(x<(k+24)).*0
+(x>=(k+24)).*(h*exp(-(log(h/b)-a*(k-c))*((24+k-c)/(c-k)))*exp(a*24)*((exp(2*a*(x-k-24)))-
1)/((exp(2*a*(x-k-24)))+1))+(x<(e+48)).*0 +(x>=(e+48)).*(d*exp(-(log(h/b)-a*(k-c))*((48+e-
c)/(c-k)))*exp(a*48)*((exp(2*a*(x-e-48)))-1)/((exp(2*a*(x-e-48)))+1))+(x<(g+48)).*0
+(x>=(g+48)).*(f*exp(-(log(h/b)-a*(k-c))*((48+g-c)/(c-k)))*exp(a*48)*((exp(2*a*(x-g-48)))-
1)/((exp(2*a*(x-g-48)))+1))+(x<(k+48)).*0 +(x>=(k+48)).*(h*exp(-(log(h/b)-a*(k-c))*((48+k-
c)/(c-k)))*exp(a*48)*((exp(2*a*(x-k-48)))-1)/((exp(2*a*(x-k-48)))+1))+(x<(e+72)).*0
+(x>=(e+72)).*(d*exp(-(log(h/b)-a*(k-c))*((72+e-c)/(c-k)))*exp(a*72)*((exp(2*a*(x-e-72)))-
1)/((exp(2*a*(x-e-72)))+1))+(x<(g+72)).*0 +(x>=(g+72)).*(f*exp(-(log(h/b)-a*(k-c))*((72+g-
c)/(c-k)))*exp(a*72)*((exp(2*a*(x-g-72)))-1)/((exp(2*a*(x-g-72)))+1))+(x<(k+72)).*0
+(x>=(k+72)).*(h*exp(-(log(h/b)-a*(k-c))*((72+k-c)/(c-k)))*exp(a*72)*((exp(2*a*(x-k-72)))-
1)/((exp(2*a*(x-k-72)))+1)))', 'independent', 'x', 'dependent', 'y' );
    opts = fitoptions( 'Method', 'NonlinearLeastSquares' );
    opts.Display = 'Off';
    opts.Lower =      [0 0 0 0 4 0 9 0 15];
    opts.StartPoint = [0.5 1 0 1 5 1 10 1 16];
    opts.Upper =      [2 Inf 1 Inf 6 Inf 11 Inf 20];

    % Fit model to data.
    [fitresult{i}, gof(i)] = fit( xData, yData, ft, opts );


    % Get parameters of model.
    coeff(i,:) = coeffvalues(fitresult{i});
end
```

Supplementary Data S3: Simulations of pharmacokinetic scenarios.

In following figures plasma profile is shown at the top left, drug concentrations in the gallbladder at the top right, percentage of drug undergoing CEH at the bottom left, and temporal increase in AUC at the bottom right.

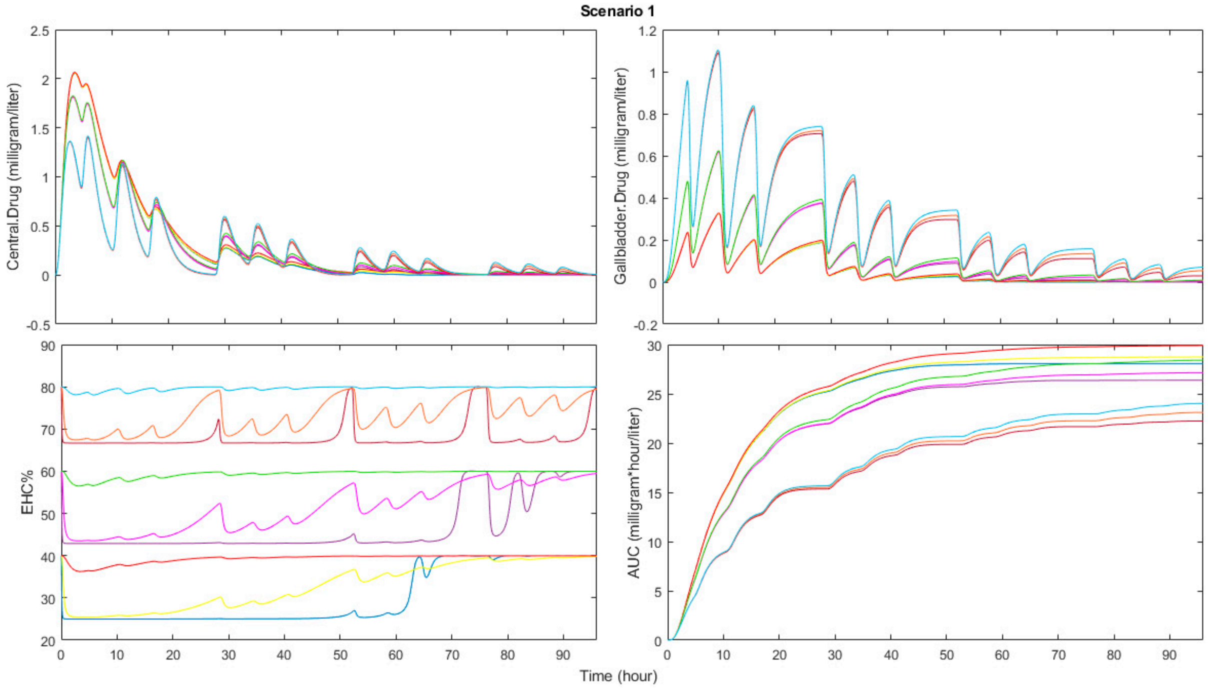

Figure S1. Pharmacokinetic simulation of scenario 1 (rapid absorption in the intestinal compartment).

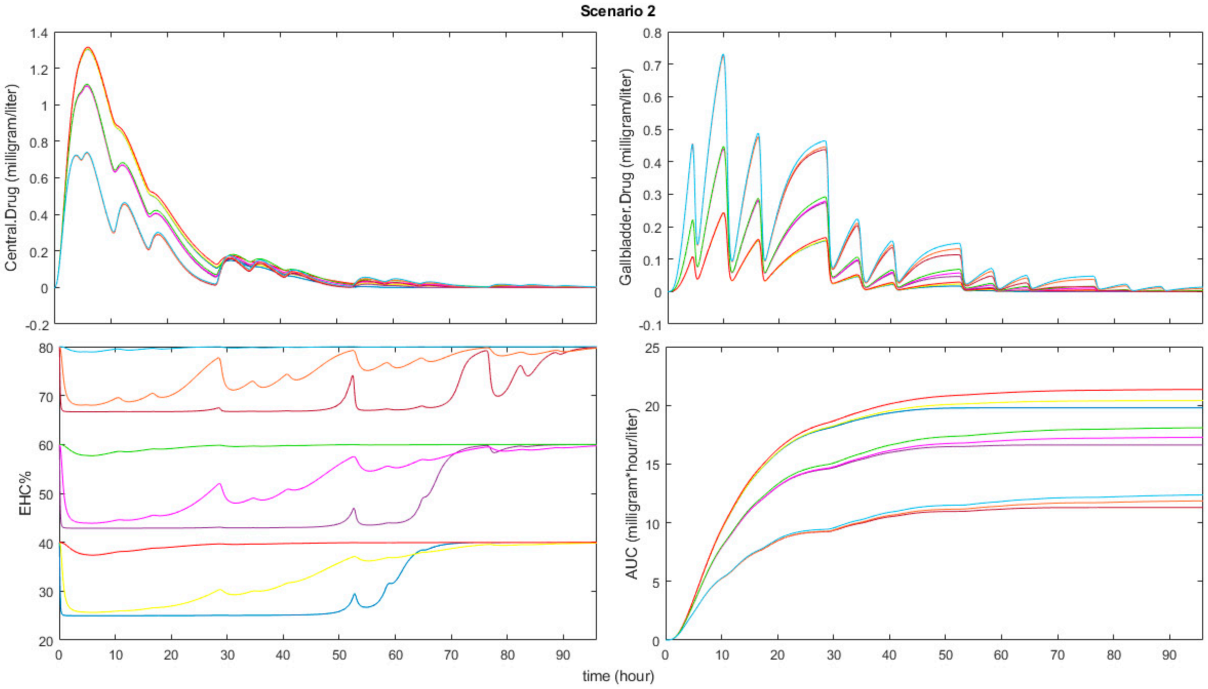

Figure S2. Pharmacokinetic simulation of scenario 2 (slow absorption in the intestinal compartment).

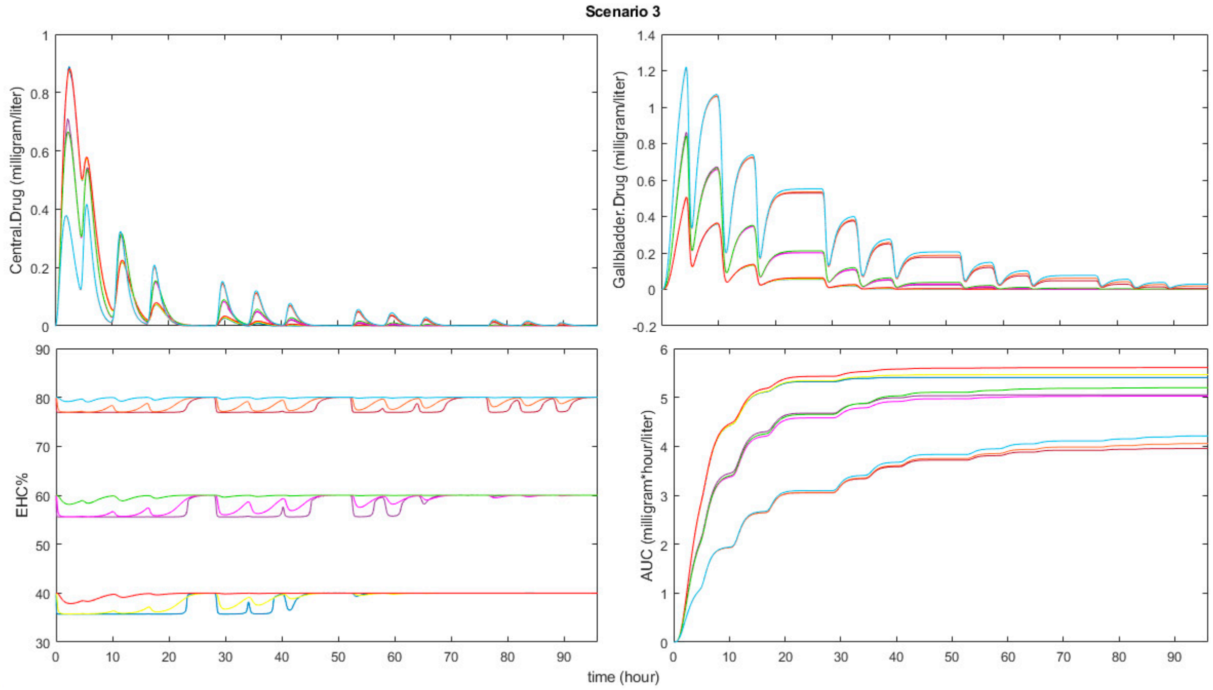

Figure S3. Pharmacokinetic simulation of scenario 3 (rapid renal elimination from central compartment).

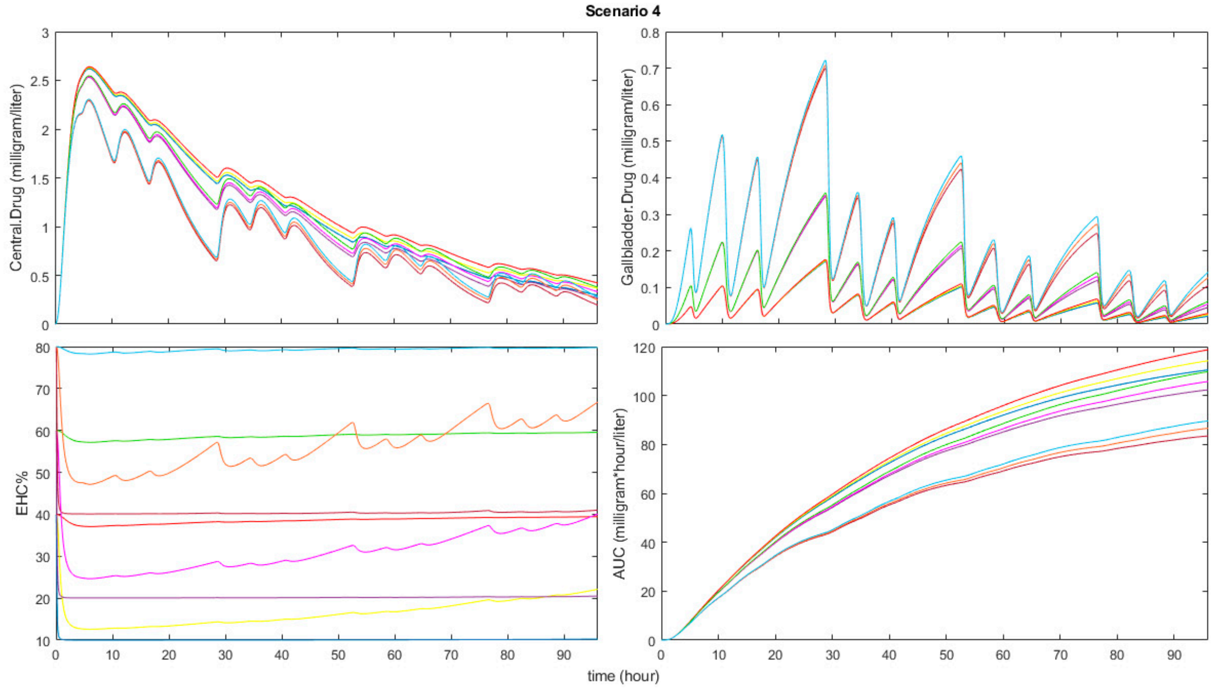

Figure S4. Pharmacokinetic simulation of scenario 4 (slow renal elimination from central compartment).

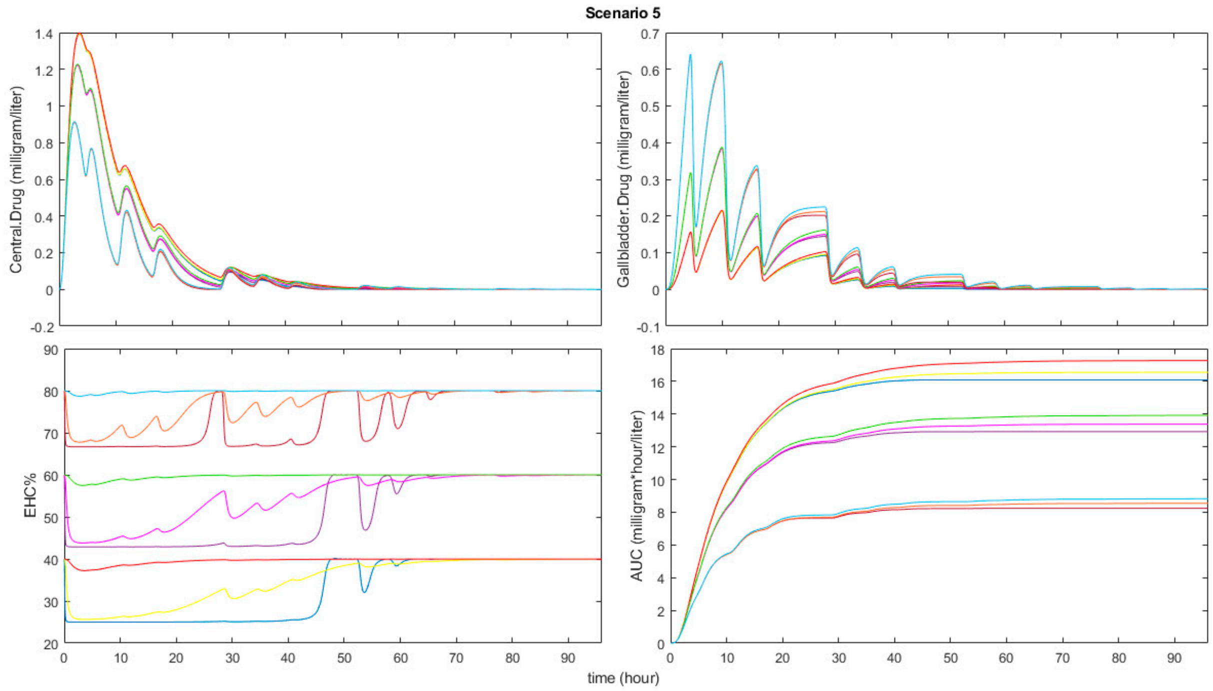

Figure S5. Pharmacokinetic simulation of scenario 5 (rapid elimination from intestinal compartment).

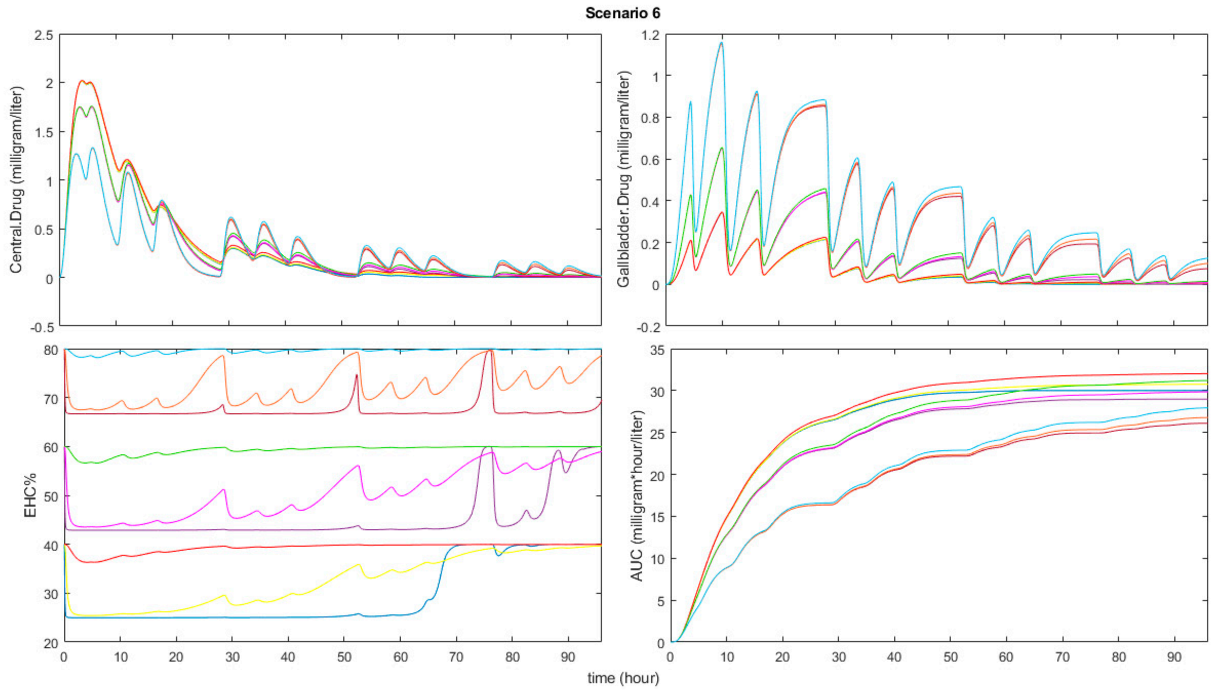

Figure S6. Pharmacokinetic simulation of scenario 6 (slow elimination from intestinal compartment).

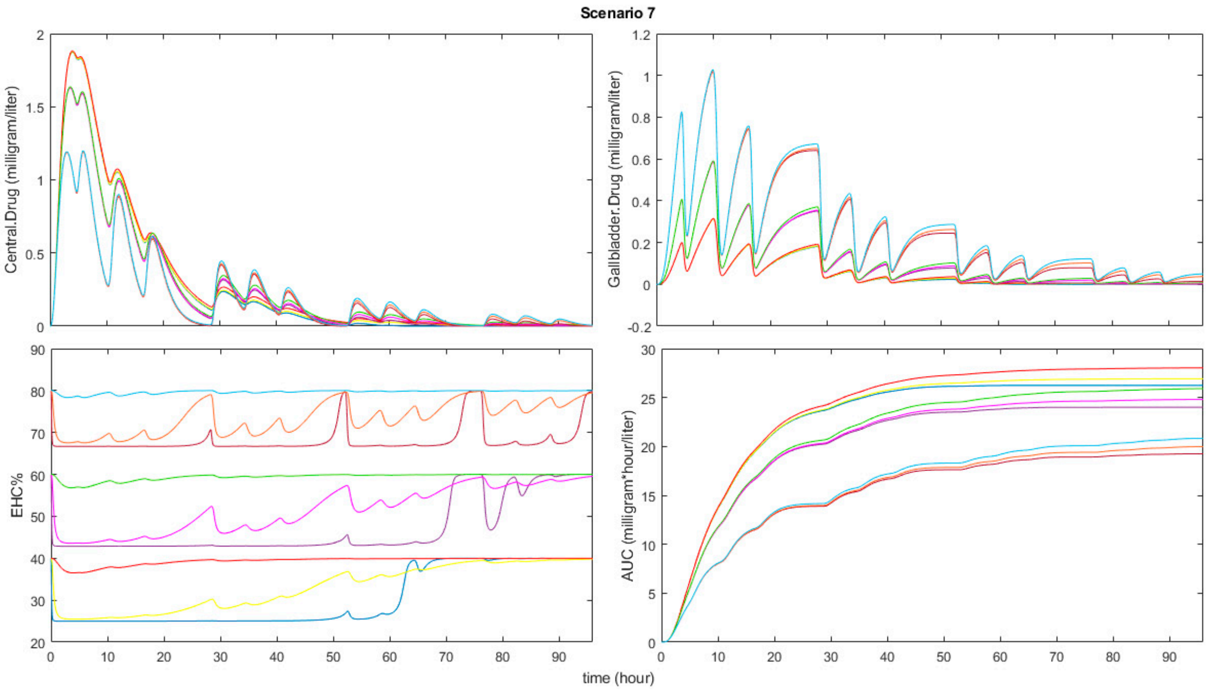

Figure S7. Pharmacokinetic simulation of scenario 7 (drug release without delay time).

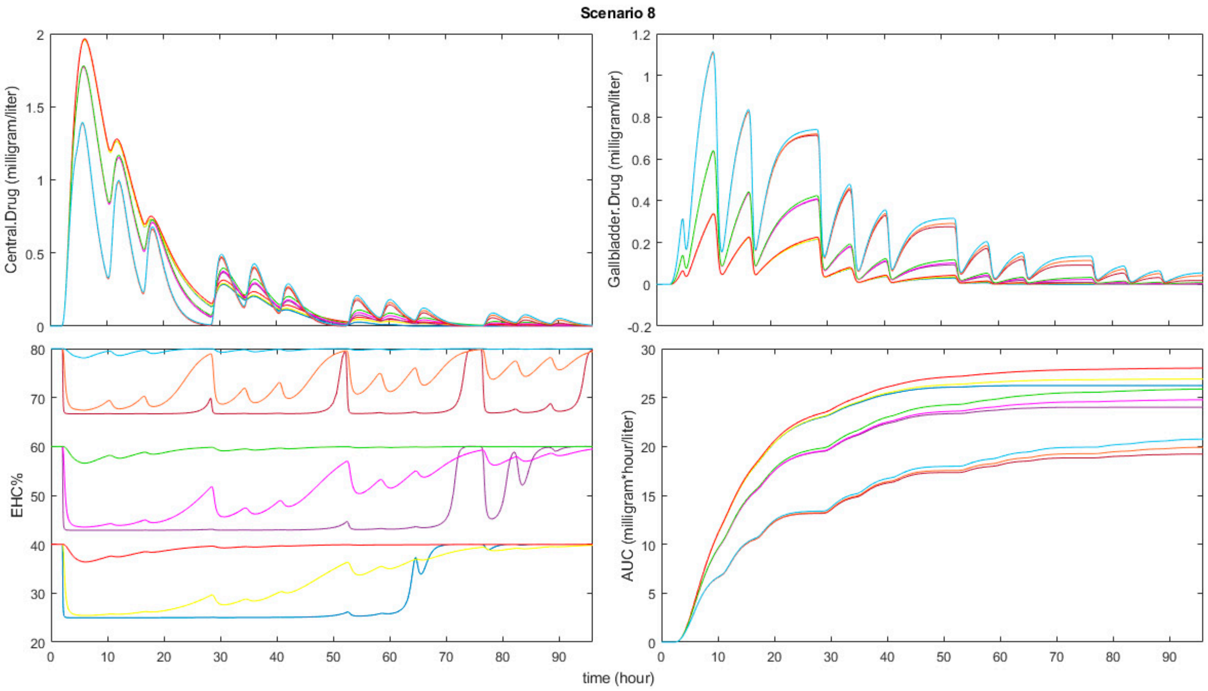

Figure S8. Pharmacokinetic simulation of scenario 8 (time-delayed drug release).

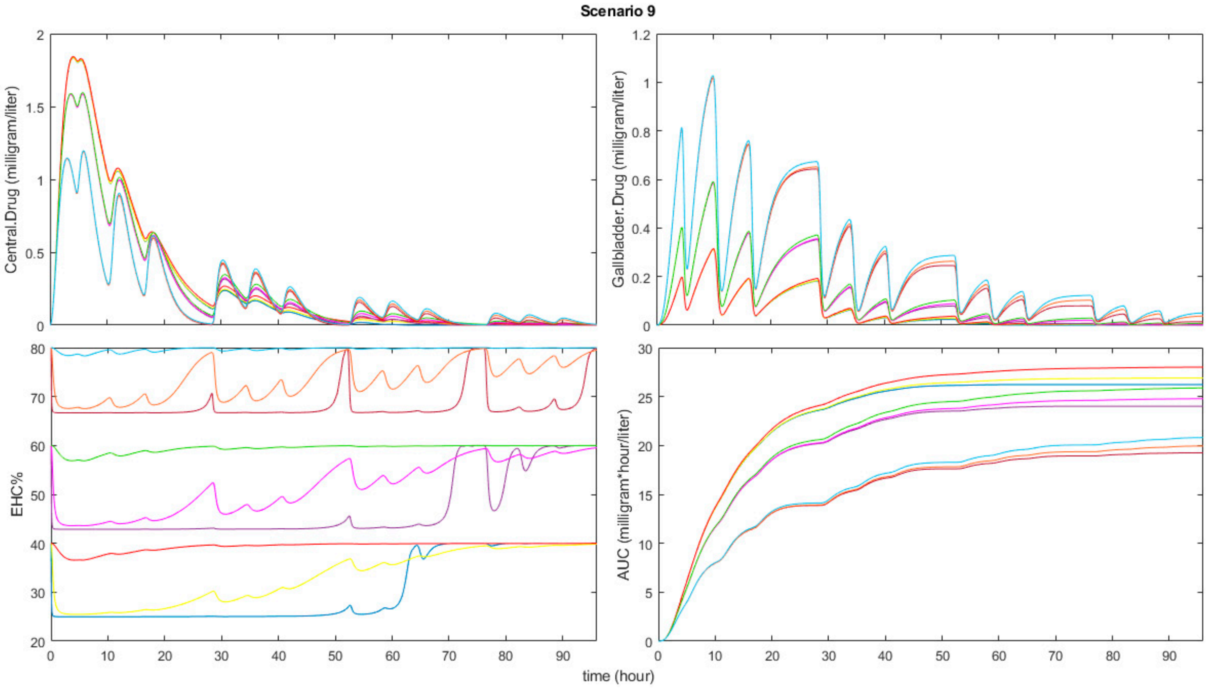

Figure S9. Pharmacokinetic simulation of scenario 9 (parabolic drug release).

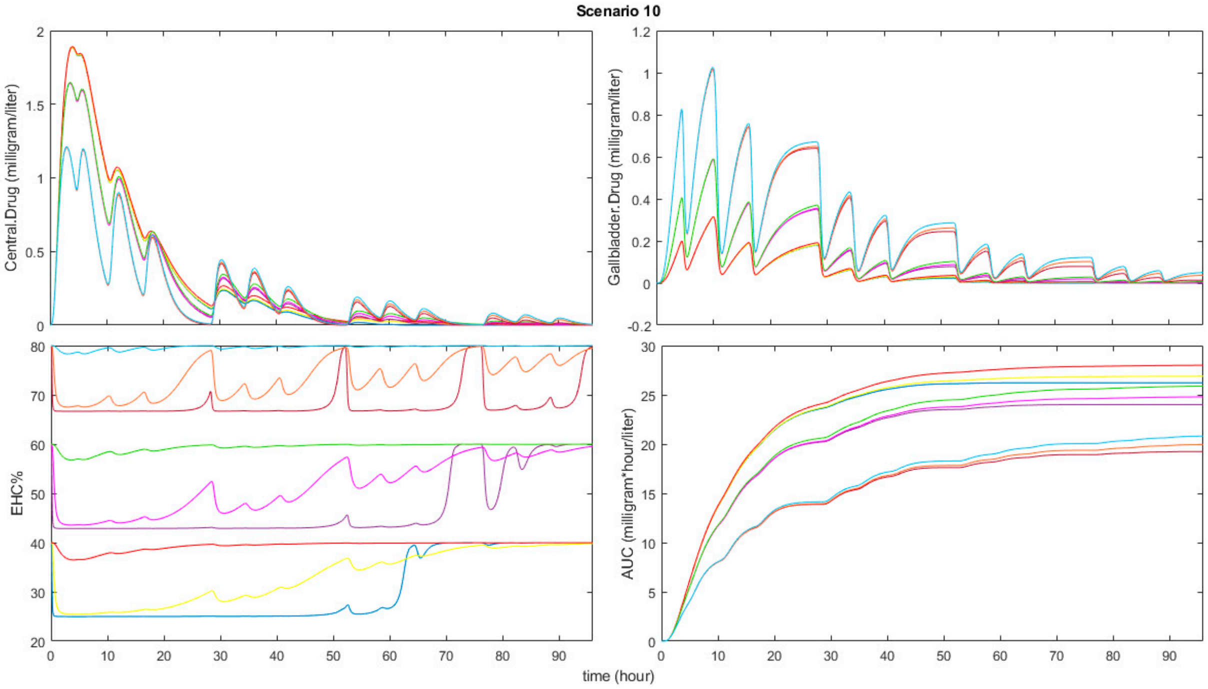

Figure S10. Pharmacokinetic simulation of scenario 10 (sigmoidal drug release).

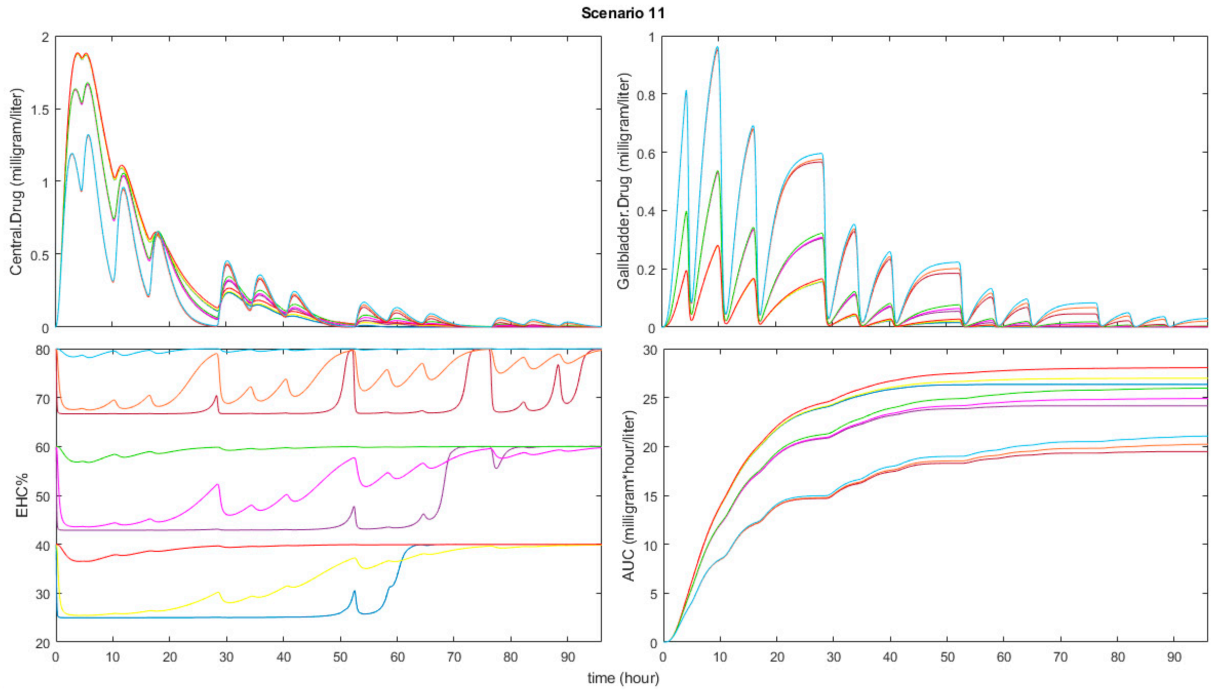

Figure S11. Pharmacokinetic simulation of scenario 11 (rapid gallbladder emptying).

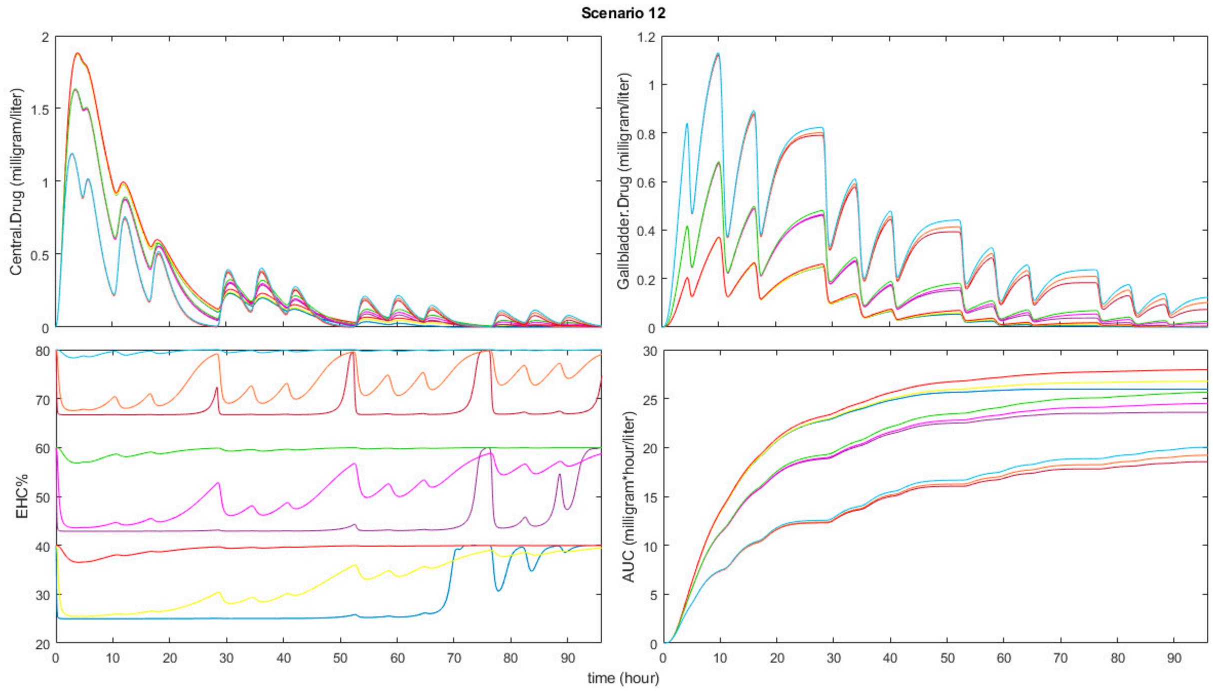

Figure S12. Pharmacokinetic simulation of scenario 12 (slow gallbladder emptying).

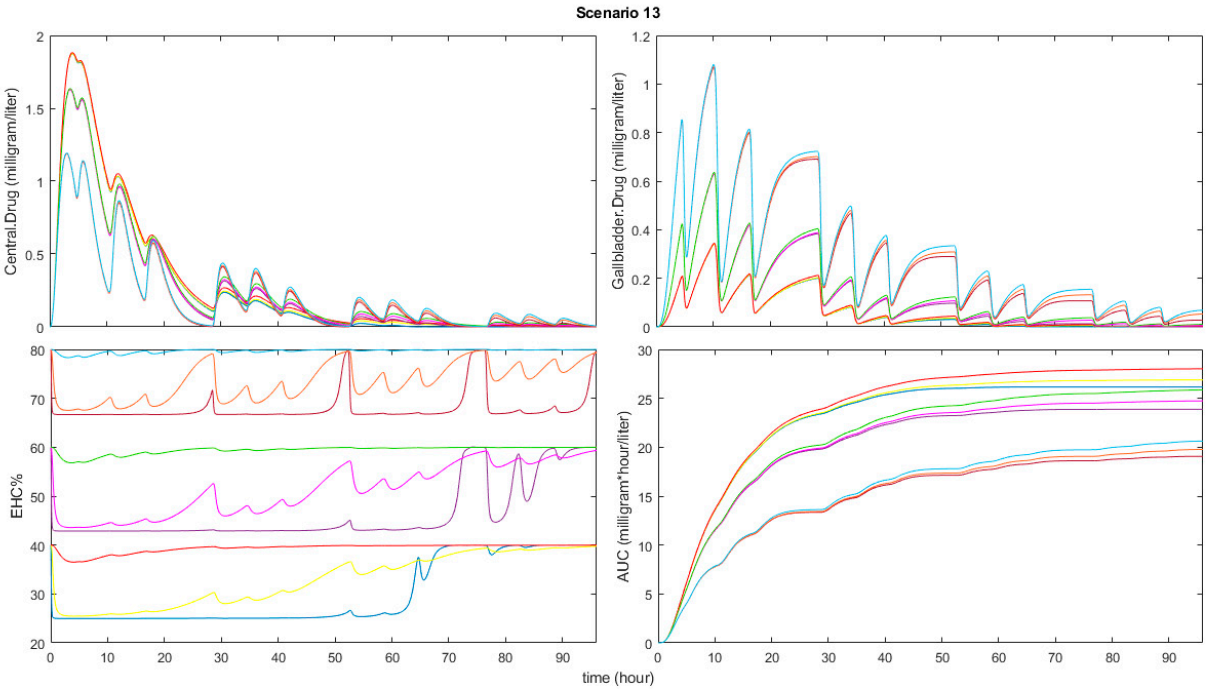

Figure S13. Pharmacokinetic simulation of scenario 13 (short gallbladder emptying duration).

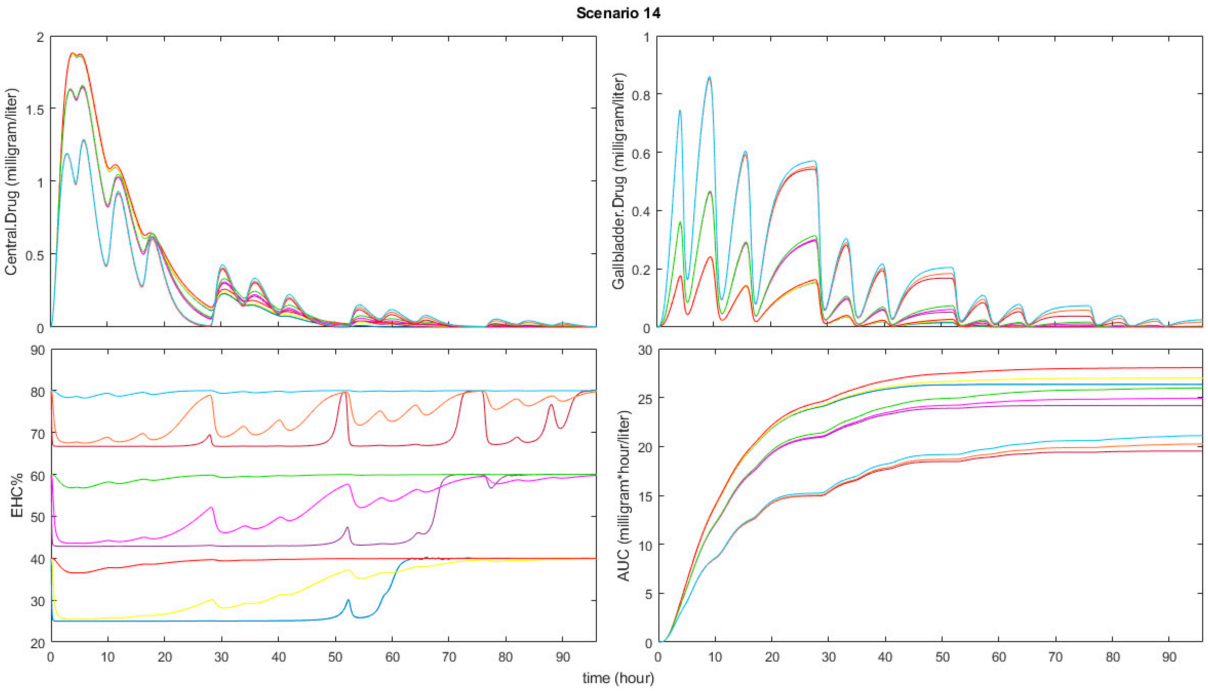

Figure S14. Pharmacokinetic simulation of scenario 14 (long gallbladder emptying duration).

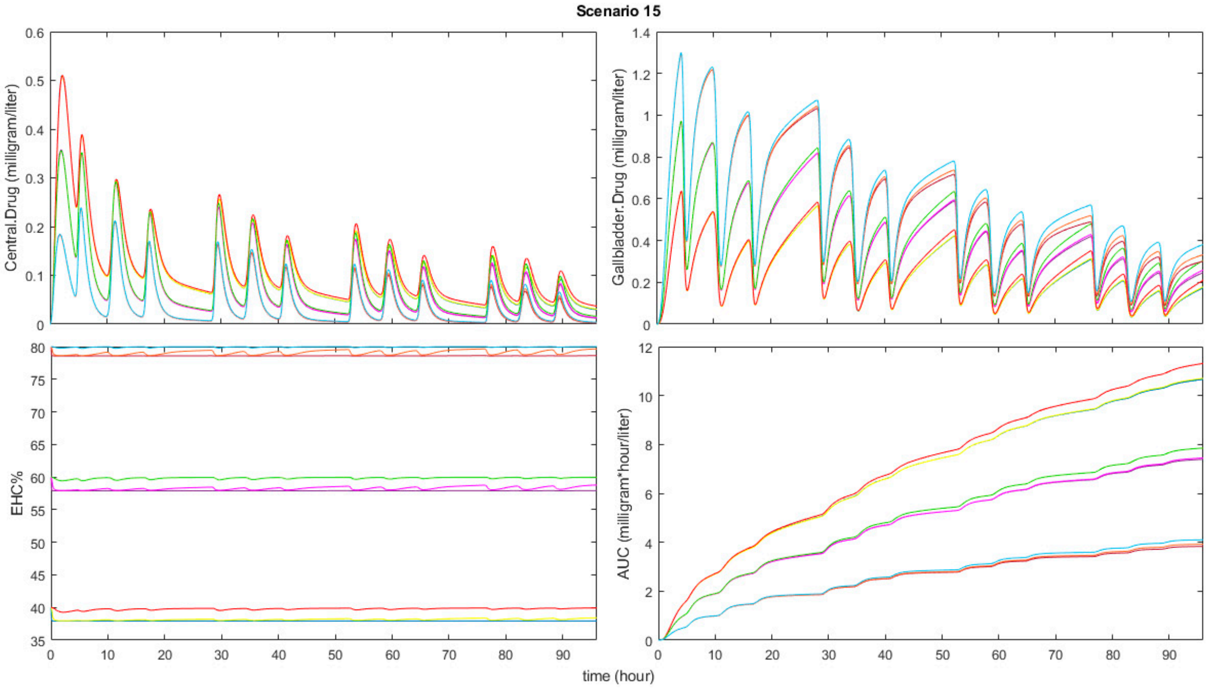

Figure S15. Pharmacokinetic simulation of scenario 15 (fast peripheral distribution with slow central return).

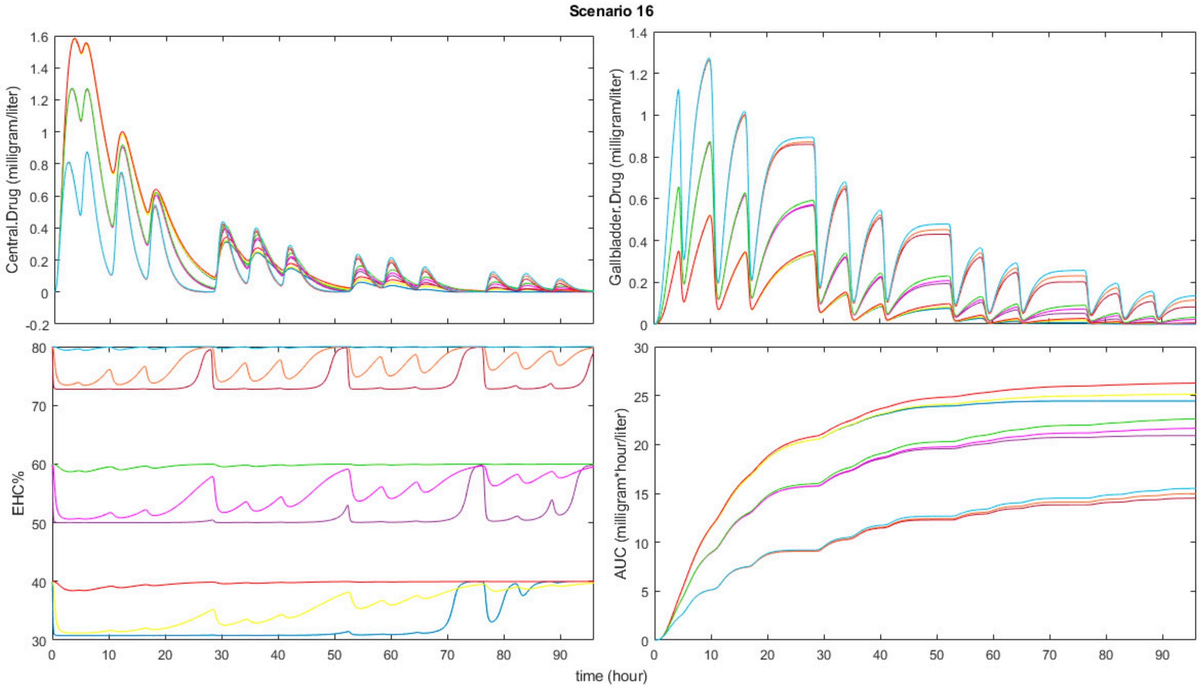

Figure S16. Pharmacokinetic simulation of scenario 16 (slow peripheral distribution with fast central return).

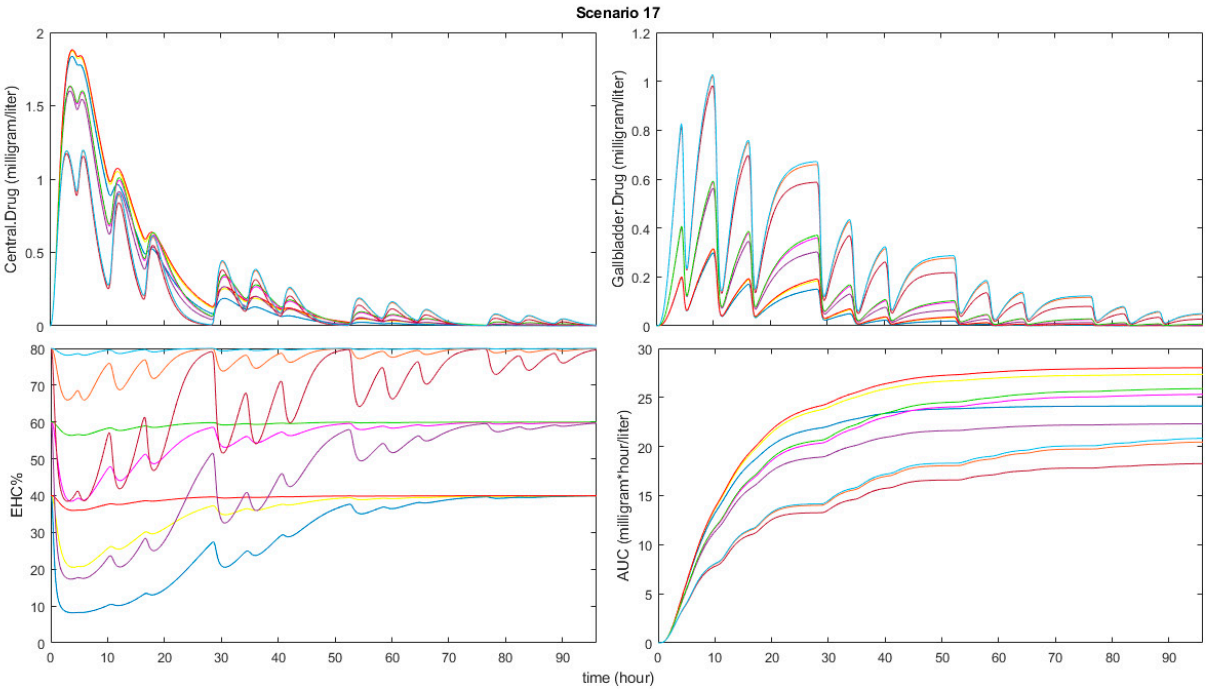

Figure S17. Pharmacokinetic simulation of scenario 17 (broad first pass elimination).

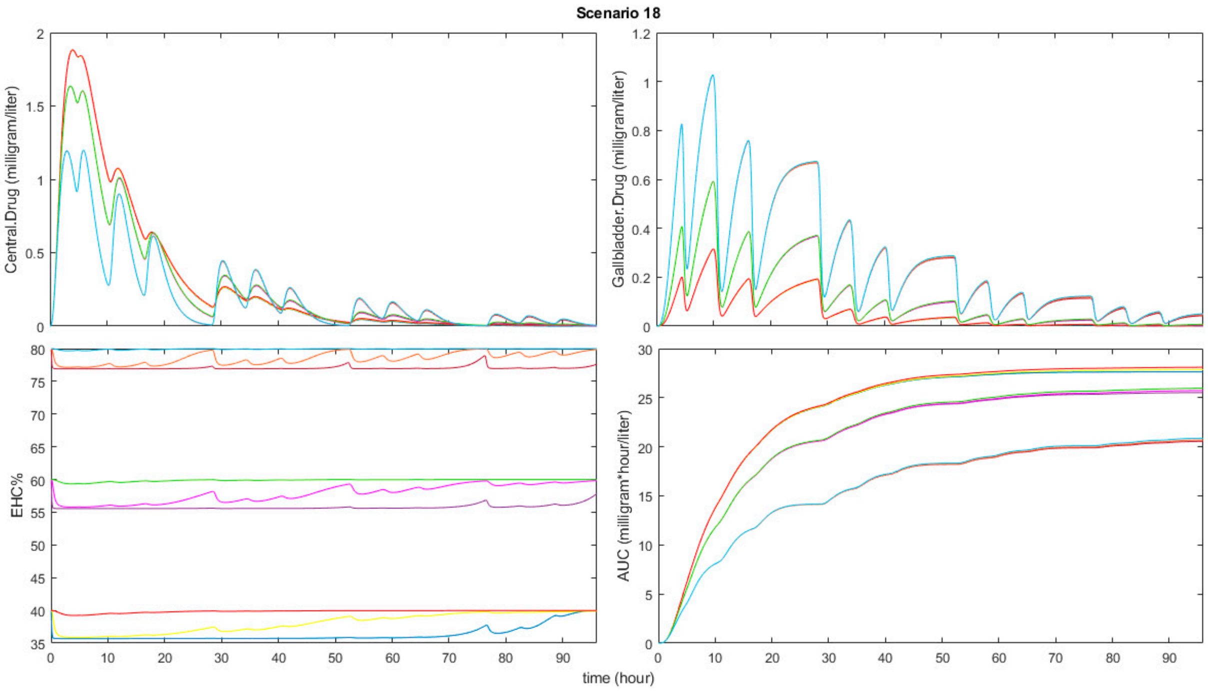

Figure S18. Pharmacokinetic simulation of scenario 18 (reduced first pass removal).

Supplementary Data S4: Fit of model M5 to selected plasma profiles.

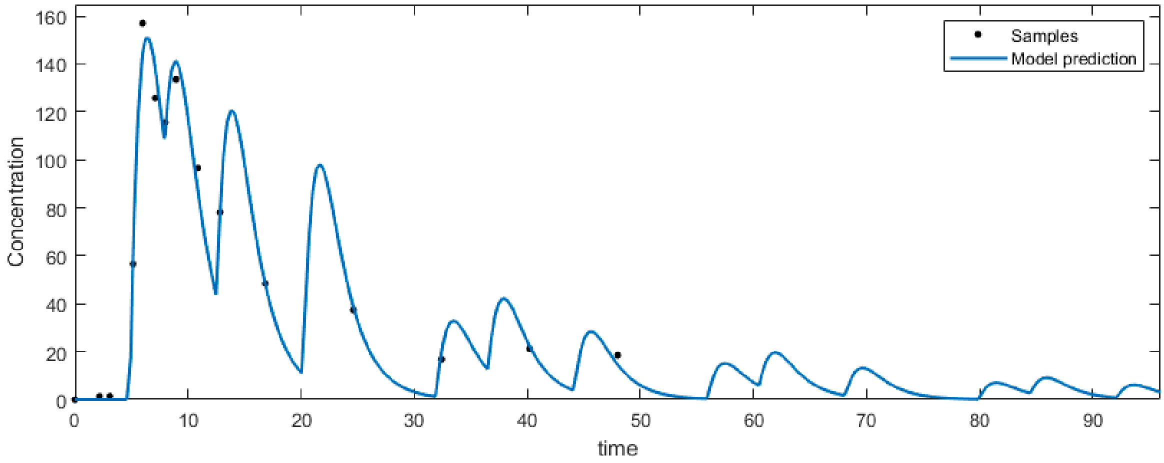

Figure S19. Fit of model M5 to plasma profile of Amiodarone.

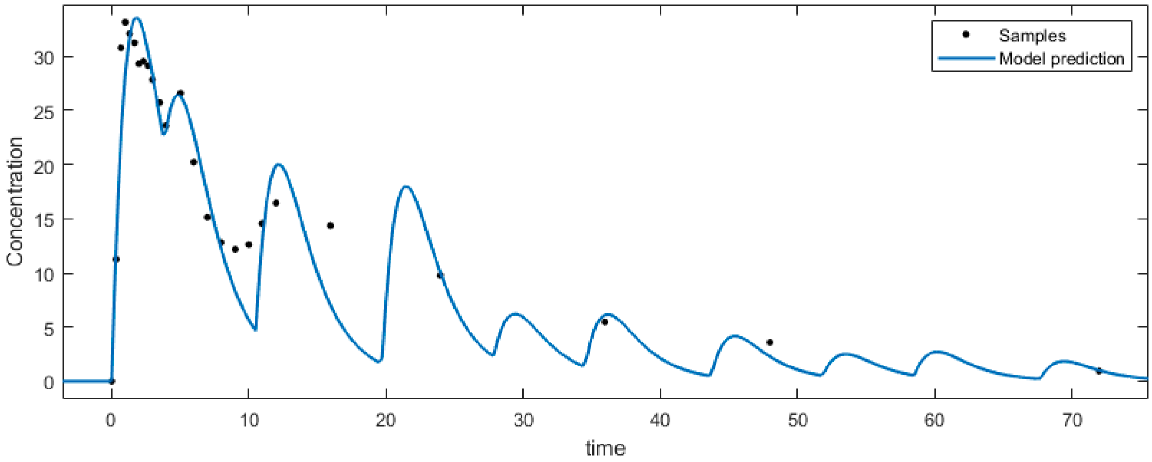

Figure S20. Fit of model M5 to plasma profile of Ezetimibe.

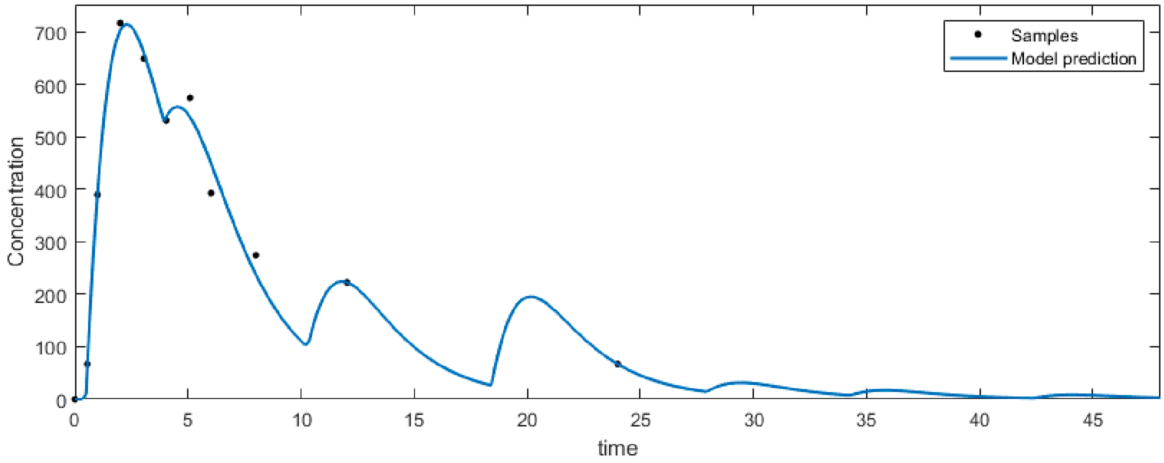

Figure S21. Fit of model M5 to plasma profile of Cyclosporine.

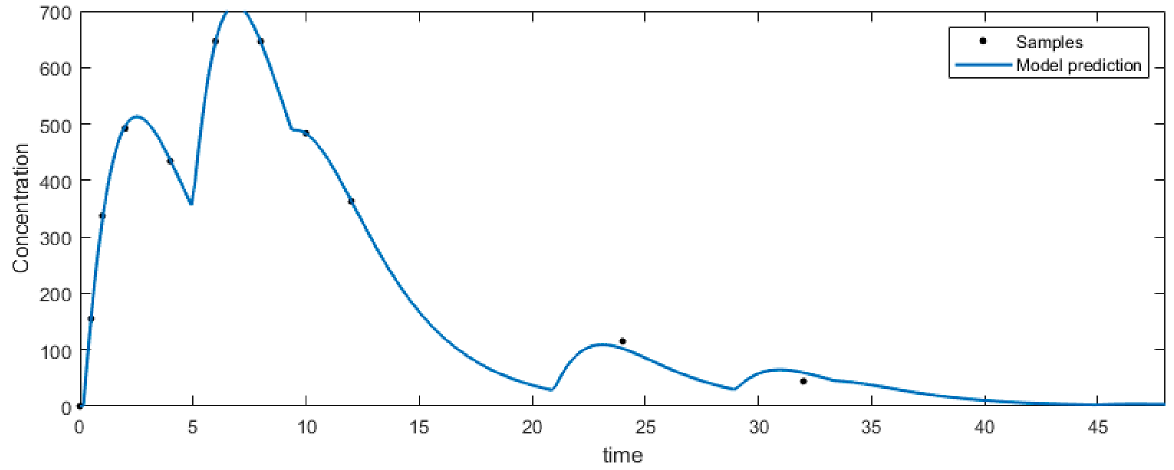

Figure S22. Fit of model M5 to plasma profile of Genistein.

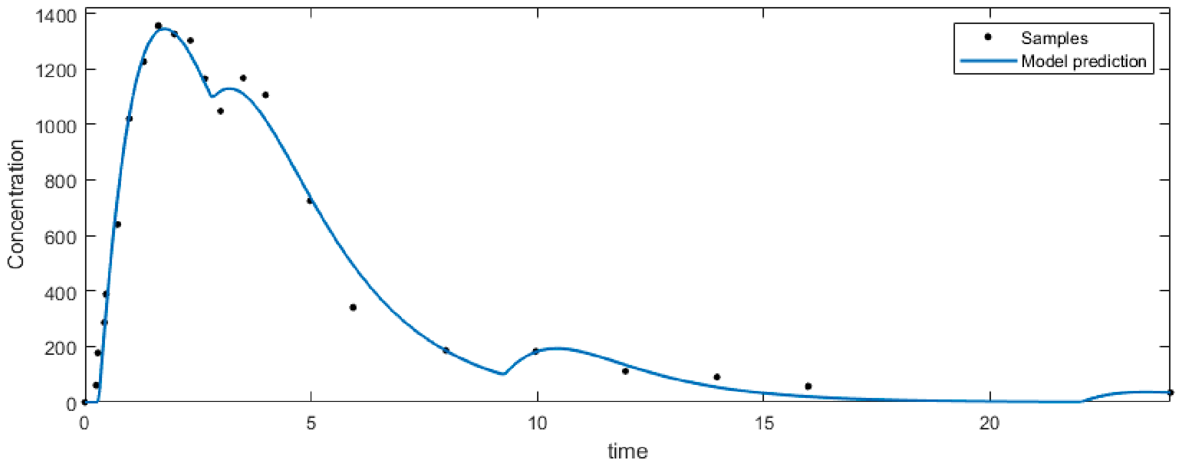

Figure S23. Fit of model M5 to plasma profile of Indomethacin.

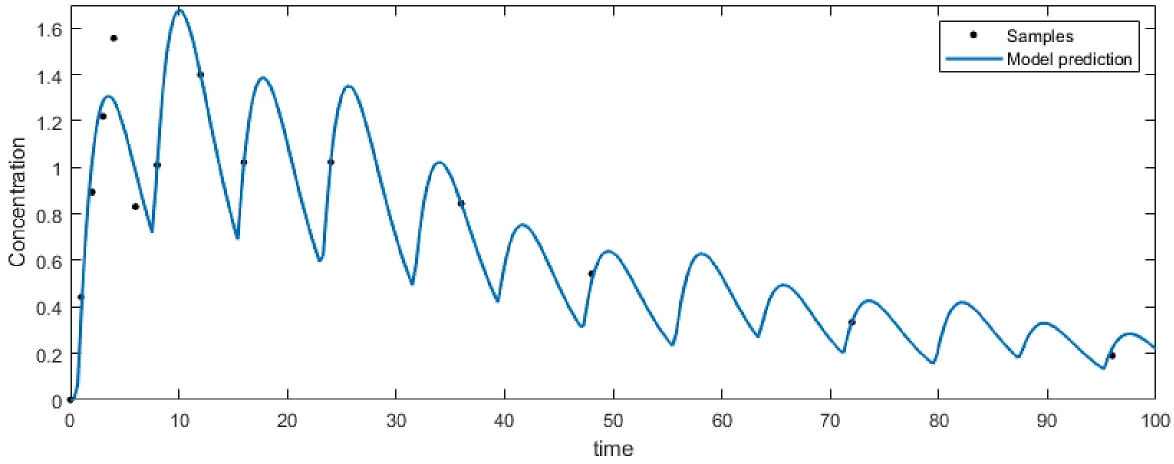

Figure S24. Fit of model M5 to plasma profile of Sorafenib.

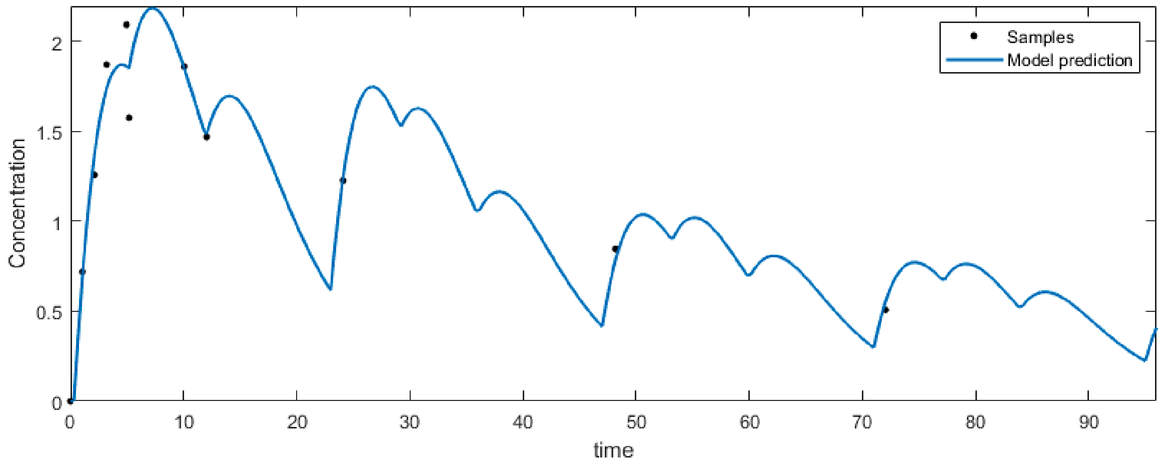

Figure S25. Fit of model M5 to plasma profile of Meloxicam.

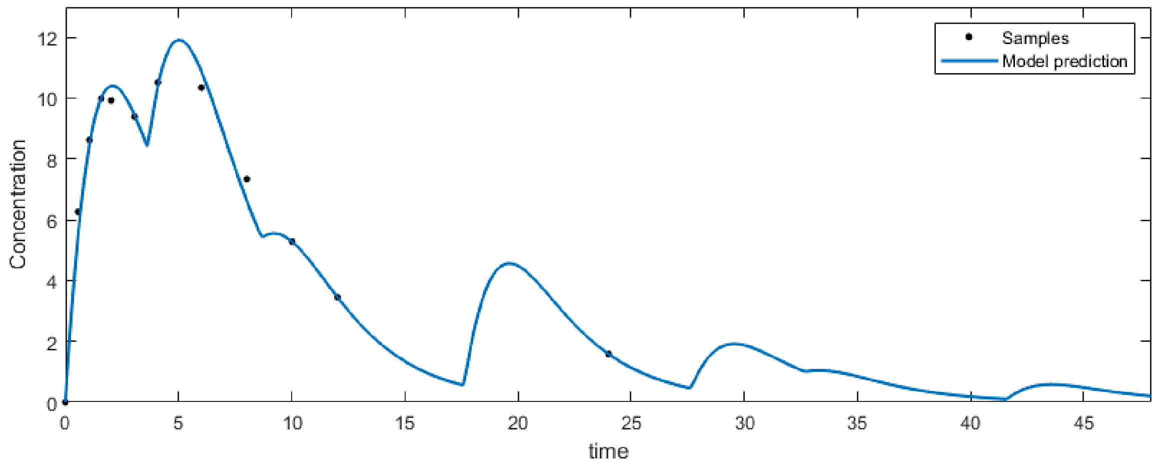

Figure S26. Fit of model M5 to plasma profile of Methotrexate.

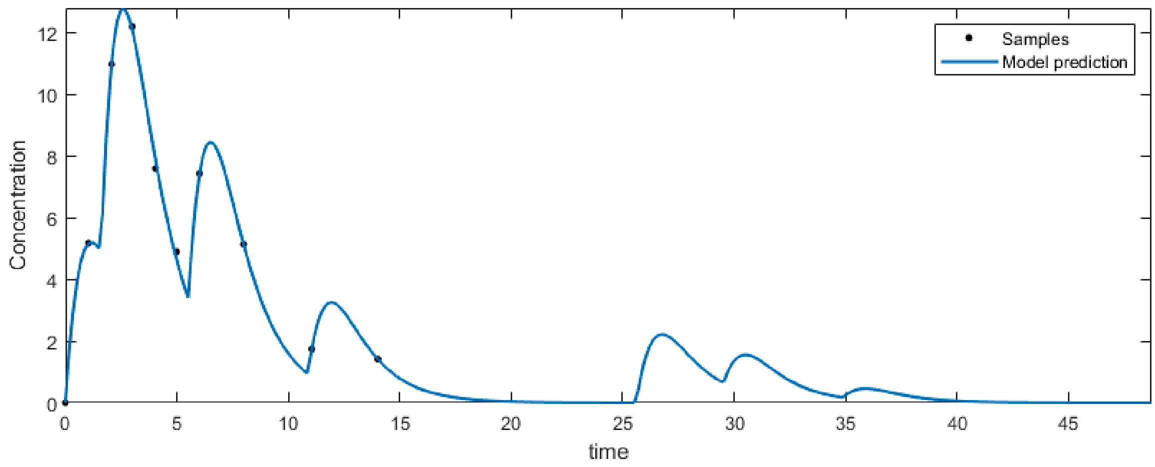

Figure S27. Fit of model M5 to plasma profile of Toremifene.

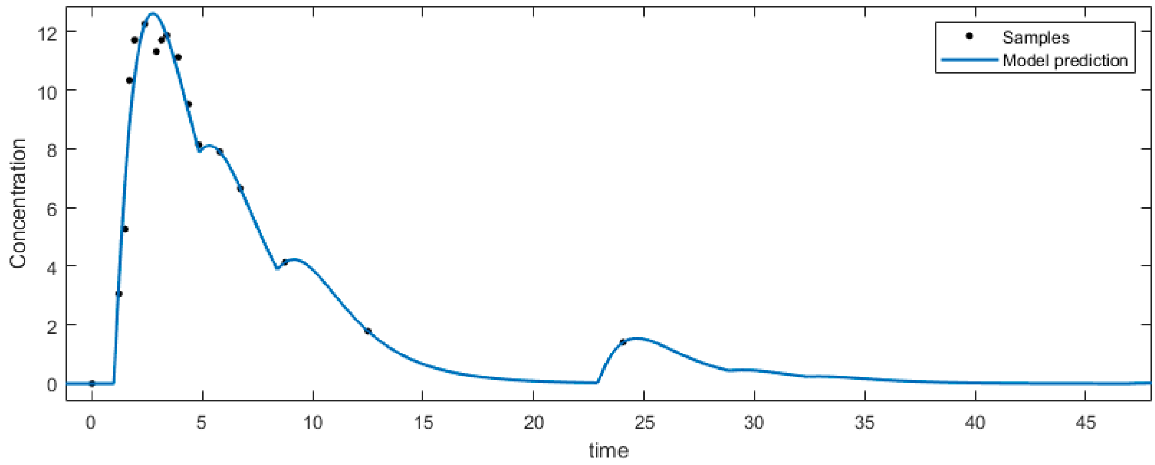

Figure S28. Fit of model M5 to plasma profile of Atorvastatin.

Supplementary Data S5: Linearity of compartmental model.

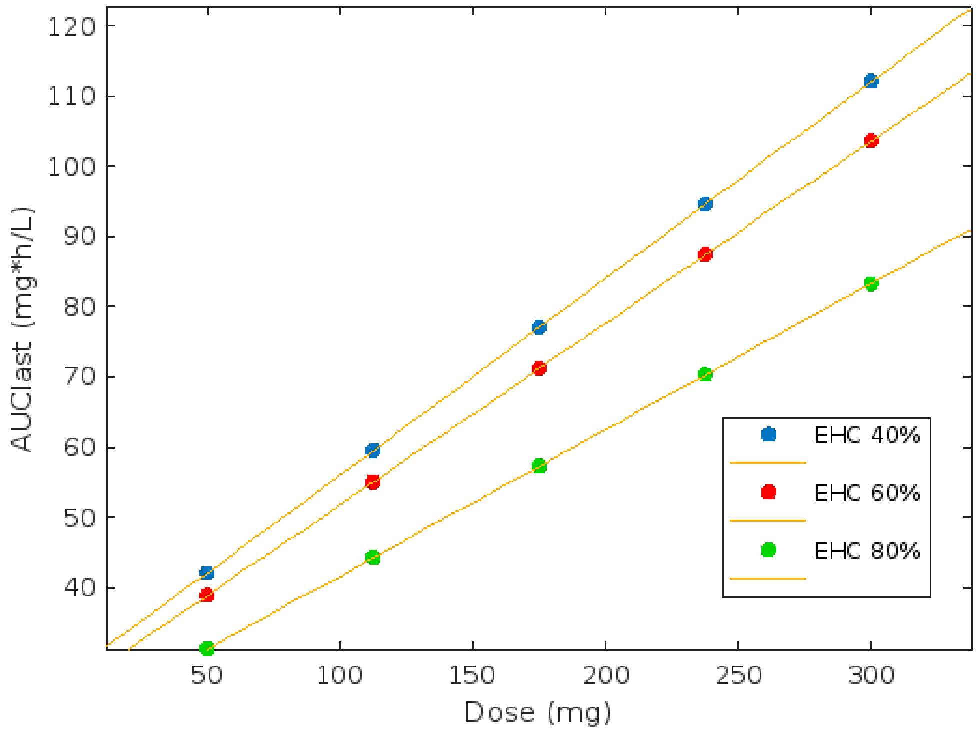

Figure S29. Linearity of AUC at different doses and degrees of EHC.
